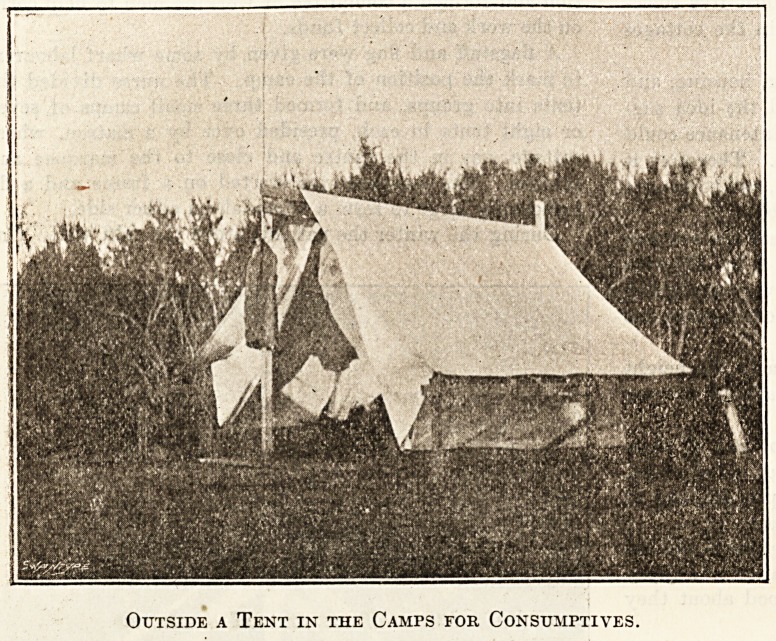# "The Hospital" Nursing Section

**Published:** 1906-04-07

**Authors:** 


### The Hospital.

### Nursing Section.

Contributions for "THE HOSPITAL," should be addressed to the EDITOR, "THE HOSPITAL" NURSING SECTION, 28 & 29 Southampton Street, Strand, London, W.C.

No. 1,019.—VOL. XL. SATURDAY, APRIL 7, 1906.

### Notes on News from the Nursing World.

### THE QUEEN AND MISS BROWNE.

ON Sunday, Miss Sidney Browne, first Matron-in-Chief of the Imperial Military Nursing Service, had the honour of being received by the Queen at Buckingham Palace. Her Majesty has throughout taken the keenest interest in Miss Browne's work as head of her service, and has graciously shown her warm appreciation of the manner in which it has been carried on. As Miss Browne is so well aware, there is no department of Army nursing of which the Queen does not keep herself well informed; and her congratulations may fitly be regarded as the crown of a most useful career.

### THE NEW MATRON OF KING'S COLLEGE HOSPITAL.

THE election of sister matron of King's College Hospital took place on Thursday last, and from a very large number of applications for the post, Miss Mary E. Ray, at present matron of Lincoln County Hospital, was chosen. Miss Ray, who thus succeeds Miss Monk, one of the most successful and most beloved of London matrons, in a position of great responsibility, at a critical time in the history of the institution, was trained at King's College Hospital, and subsequently served for four years as ward sister. The esteem which she earned in that position augurs well for her return to London as chief of her old training school. She served for three years as assistant superintendent at Leeds General Infirmary, one of the largest training schools in the provinces; and for the last seven years she has been matron of Lincoln County Hospital. At Leeds Cottage Hospital Miss Ray is most pleasantly remembered, and the matron and staff at the General Infirmary are gratified by her appointment to King's College Hospital. But, of course, her chief work has been at Lincoln, where her thorough knowledge of all branches of the work of a matron, and her gentleness, patience, and tact have made her an example to those with whom she has come in contact, whilst she did much to brighten the lives of the patients and to help and guide those who have worked under her. During the seven years of her matronship at Lincoln many improvements have been made in the nursing and domestic arrangements of the hospital. A laundry has been built, and also a new theatre, which is fitted with all the latest improvements and is most complete in all its details. A ladies' guild for providing household linen, etc., for the hospital has been started, and in the two years of its existence it has already added the equivalent of £400 to the funds. The work of the hospital has very largely increased in every department, and the nursing staff, under Miss Ray's able supervision, has kept well abreast of the times. Courses of lectures to the nurses are regularly given by members of the medical staff, with practical demonstrations on nursing by the matron. Before receiving their certificates on the completion of their three years' training, the nurses have experience in all branches of the work—medical, surgical, out-patients, and theatre—and their training has been sound and thorough. Miss Ray's own influence and character, and her thorough professional knowledge, have made themselves felt on all sides, and those who have left Lincoln look back upon the many lessons they learnt during their training, and, above all, to their connection with Miss Ray, with deep gratitude. It is with the most sincere regret that the County Hospital parts with so popular a matron, but she will carry with her the love and gratitude of many in Lincoln and the earnest wishes of all for success in her new sphere of work. She takes up the reins at King's in the prime of life and in the fulness of her powers. We feel convinced that an excellent appointment has been made.

### THE ANNUAL MEETING OF THE PENSION FUND.

IN consequence of the absence of Mr. Everard A. Hambro, Chairman of the Royal National Pension Fund for Nurses from England, the annual meeting of members will not take place until June 6. The usual notice will be issued in due course, and the Council cordially invite policy-holders to attend. It is hoped that as many as possible will be present. A preliminary statement of the results of the past year's work has already been issued, from which it is gratifying to learn that at the close of 1905 the invested funds reached the huge total of one million sterling. The number of policies issued during the year was 1,300, a greater number than in any previous twelve months of the history of the Fund. Sick-pay amounting to £1,800 was distributed, and in the last quarter of the year pensions were being paid at the rate of over £14,000 per annum. The total income of the Fund from all sources now considerably exceeds £140,000 a year.

### REMARKABLE WORK OF A MIDDLESEX NURSE IN NEW ZEALAND.

THE graphic account which appears in our columns of to-day of the construction of camps for consumptives in New Zealand will be read with general interest. We have great pleasure in stating that the nurse who is entitled to the entire credit for the initiation and extension of this most useful movement at the Antipodes is Miss Sibyl Maude, who was trained at the Middlesex Hospital. Going out to New Zealand, she acted as matron of the Christchurch Hospital for three years. For the past nine years, however, she has been doing district nursing in the town, and it was the difficulty of not knowing what to do with consumptives that suggested to her the possibility of founding a camp. She thinks that it would be possible for something of the same kind to be tried in England, and, at all events, the information furnished in the article proves that it is possible to combine efficiency with economy. One obvious and special advantage of the tents and shelters in use at Christchurch is that they can be easily moved on to fresh ground when necessary. It should be added that in New Zealand the title of "Nurse Maude Camps" is very appropriately given to this notable practical outcome of individual knowledge, enterprise, and energy.

### NEW QUARTERS FOR TOTTENHAM NURSES.

The extensions now going on at Tottenham Hospital include two new wards containing ten beds each, which, with alterations to old wards, will bring the number of beds up to 120. An important addition has also been made to the administrative block, including twelve nurses' bedrooms. A large room is being remodelled as a nurses' dining-room, their former one having been swallowed up in a new ward; airy corridors now connect one part of the hospital with the other; a new lift for patients is just completed, and an outside iron staircase provides means of escape in case of fire. For months an atmosphere like that of a new and enlarged edition of the annual spring cleaning has pervaded the hospital. Rooms, cosy and comfortable one day, are completely dismantled the next, and become something quite different in a few weeks' time. The nursing staff are awakened in the mornings by hammering, sawing, and uncouth noises of every kind, and are lulled to sleep at night by weird sounds from distant engineers and "fitters" working "overtime." They hail the comparative quiet of each week-end with a fierce joy known only to those in like circumstances, and Saturday afternoon is the one time in the whole week when the maids really put any heart into the cleaning of corridors and stairs.

### NURSING IN THE EIGHTIES.

It is not a very far cry to the eighties, but a contributor who in another page recites her experiences at Sheffield in that period, supplies the nurses of to-day who grumble about trifles, with wholesome matter for reflection. Most of these who take the trouble to compare their lot with that of the staff at Sheffield Infirmary a quarter of a century ago, will find solid cause for congratulation. What would the nurse who complains of having to eat the same kind of meat two days in succession say to beef or mutton every day with a pudding only on Sunday? Or the nurse who protests because she has only half an hour for her meal in a well-appointed dining-room, think of being compelled to go dinnerless because she had no time to cook a raw chop and the potatoes which she found in her bedroom? The modern night nurse, under present conditions, frequently considers herself overworked, but how would she describe herself if she had 70 patients to attend to, women and children, medical, surgical, and ophthalmic?

### THE GARTLOCH NURSES AND PARLIAMENT.

In the House of Commons last week Mr. Barnes asked the Secretary of State for Scotland "whether he is aware that the Glasgow Parish Council have approved of new regulations for nurses in their employ whereby the said nurses had their average weekly hours of labour increased to 71, and their maximum salaries reduced to £35; and seeing that the Exchequer gives maintenance grants towards those nurses' upkeep, does the Government intend to sanction the new regulations." The reply of Mr. Sinclair was to the effect that the Government has no power to interfere either with the regulations or with the present distribution of the grants. "The regulations," he added, "will not come into force for some weeks, and a petition has now been presented to the Council by the nurses asking that they may have each alternate Sunday off duty, which, I am informed, will meet their wishes."

### "CHURCH TIME."

A somewhat unusual request has been made by the nurses at Wolverhampton Poor-law Infirmary. They have asked the Guardians to allow them time to worship in churches outside the workhouse. There is a church in the institution available, but this does not appear to meet the case. The request has been referred to a committee. We think that it should be easily possible to permit a certain number of the staff to attend church outside the workhouse in turn. On such questions as this every reasonable effort should be made to comply with the wishes of the nurses.

### AN OPERATION CASE.

At the last meeting of the Guardians of St. George's, Hanover Square, a report from the Infirmary Committee was read recommending that, subject to the approval of the Local Government Board, a fee of ten guineas should be paid to Dr. L. A. Bidwell, without prejudice, for performing an operation upon a probationer nurse who was suffering from a perforated ulcer of the stomach. The usual fee for such an operation, it was pointed out, was thirty guineas, but as there was no precedent for the payment of so large a fee by the Guardians, the Committee were only able to recommend the payment of ten guineas, it being explained to Dr. Bidwell that this sum was not offered as adequate renumeration, but in grateful recognition of his valuable and efficient services. It was unanimously decided to pay the amount, and we have no doubt that the Local Government Board will give their sanction to the very proper course pursued.

### THE IMPERIAL MILITARY NURSING SERVICE.

We are officially informed that Miss. D. M. Taylor, sister in Queen Alexandra's Imperial Military Nursing Service, has been transferred to Egypt from the Royal Infirmary, Dublin. Miss A. B. Cameron, staff nurse, has been transferred to the Queen Alexandra's Military Hospital, Millbank, on her return from South Africa. Miss A. J. St. Clair and Miss A. M. Phillips have been posted to the Royal Victoria Hospital, Netley; Miss D. M. Smith to the Military Hospital, Portsmouth; and Miss G. A. Aitchison to the Military Hospital, York, on their appointment as staff nurses. Miss A. E. FitzGerald, staff nurse, has been transferred to the Military Hospital, Gibraltar, from the Military Hospital, York; Miss K. M. Bulman, staff nurse, to the Military Hospital, Malta, from the Military Hospital, Portsmouth; and Miss M. E. Wilkin and Miss A. M. MacCormac, staff nurses, to the Military Hospital, Malta, from the Royal Victoria Hospital, Netley.

### CHANGES AT A LIVERPOOL HOSPITAL.

There have been further changes at the Royal Southern Hospital, Liverpool. On March 1 Miss Sproule retired after more than a quarter of a century's service in the institution—for many years as sister and home sister, and for the last three as matron. Miss Sproule was presented by the Committee and Medical Board with a silver inkstand and a purse of 100 guineas, and by the resident staff with a gold watch, one and all wishing her much happiness in her well-earned rest. The Committee have again chosen one of their own staff to succeed Miss Sproule as head of the training school. Miss Williams has also been connected with the hospital for several years as sister of the Albert Ward, and for the last three years as home sister, and the whole staff are pleased that she has been promoted to the position of matron. Miss Hadfield succeeds Miss Williams as home sister, and this appointment has also been received with satisfaction by the nursing staff.

### SICK-ROOM COOKERY AT CROYDON INFIRMARY.

The examination in sick-room cookery has recently been held at Croydon Poor-law Infirmary, the result being most satisfactory. All the nurses passed "First Class," the maximum number being 60. Nurses Francis K. Pattenden, Eva Marie Cahill, Marian Humfrey, Rhoda Tadgell, Sarah Roberts, Annie L. Stevens, Ethel L. Searle, Kate McKeon, and Madaline Bellamy, secured 59 marks; Nurses Mary Thompson and Mary Harvey, 58; Nurses Katherine Godfrey and Florence E. Whitehouse, 57; and Nurse Lois Ellen Hulme, 53. Their instructress, Miss Baker, who took great interest in teaching them, is to be congratulated upon the results.

### HOME FOR MOTHERS AND BABIES.

In the first annual report issued in connection with the Home for Mothers and Babies and Training School for District Midwives, at Woolwich, opened in May last year, Miss Alice Gregory, the hon. secretary, furnishes on behalf of the Committee of Management, some general details of the events of the period. Dr. Mary Roche deals with medical matters, and Mrs. Parnell, the lady superintendent, with the nursing. It appears that 87 patients have benefited since the Home was opened, 61 in the wards, and 26 in their own homes. The number of cases treated each month steadily rises, and in order that the Home should not be dangerously overcrowded, it has become necessary to urge patients, when possible, to enter their names at least two months before their confinement. There were four pupil midwives in training at the end of the year, and plenty of other candidates only deterred from entering upon training by inability to pay the fees. To relieve this difficulty two scholarships have been given for the present year.

### SUNDERLAND POOR-LAW HOSPITAL.

In recommending that the usual certificate be granted to six probationer nurses at Sunderland Poor-law Hospital, the examiner specially commended Nurse Dinsmore, who was not only excellent in every branch of the examination, but gained 95 per cent. of the marks allotted. Dr. Modlin added in his report that "if all the nurses are as well up in their work as these six whom I have examined, the House Committee are to be congratulated upon their nursing staff."

### CONVICTION FOR THEFT.

On Saturday, at Bow Street Police Court, the Magistrate sentenced Alice Nicholls, a nurse, to two months' hard labour for stealing a case containing jewellery belonging to Miss Elizabeth Camm, also a nurse. Both were living at the Nurses' Hostel and had become friendly, until Miss Camm found that her jewel-case and the contents, including two brooches, two rings, a watch, and a scarf pin, had disappeared from her trunk. The evidence of a pawnbroker's assistant was conclusive, and, practically, no defence was attempted. It is very sad that a nurse should fall so low, but the prosecution was essential, if only as a matter of justice to the other residents of an exceedingly well-conducted establishment.

### REST FOR TIRED NURSES.

The importance of providing accommodation for nurses needing rest and recuperation has been recognised in this country, and we see that an effort in the same direction is being made in America under the auspices of Mrs. Milligan, at Las Vegas, New Mexico, who, as Miss Latter, was formerly head nurse at St. Andrew's Infirmary, New York City. In 1898 her husband, Dr. Milligan, established a sanatorium, and in connection with this institution his wife is now starting a nurses' department for nurses in want of rest "at such a price as to be within the means of almost any one." As a matter of fact, the charge is thirty dollars per month, any medical treatment required being gratuitous. In the old country, many tired-out nurses get a period of rest without any charge—as at Parkwood, Henley-on-Thames, which has just been closed to nurses but will be open to them again in the autumn—but those in the United States who can afford to pay will doubtless appreciate this new departure.

### The Nursing Outlook.

"From magnanimity, all fears above;
From nobler recompense, above applause,
Which owes to man's short outlook all its charm."

### HOLY WEEK.

Workers amongst the sick must in large measure enter into the spirit of Holy Week, and so renew their strength. The mystery of suffering and pain often strikes the least observant of our race, and the meaning of both is sometimes revealed in some measure to the practitioner and the nurse. At their best illness and suffering result in the renewal of spiritual life. They entail an enforced period of abstention from active life in the world, and so enable each one of us to consider our lives and to take stock, as it were, of the results which may have accrued from them to others as well as to ourselves. Few things are more helpful to the observant than the patience and resignation of a good man or woman in the hours of suffering. It is wonderful to realise how much solid goodness there is often in the average man or woman if we can only reach it. Illness softens character, and very often reveals its best attributes, and so the nurse, who is also a good woman, gains strength for her work from association with many of her patients, and to her as to them Holy Week sometimes marks the attainment of greater strength and nobility of purpose in the daily life.

There are two aspects of Holy Week which we may usefully consider in connection with the sick and those who minister to their necessities. In the present day nothing has been more remarkable than the power which has resulted from co-operation amongst bodies of workers. Such co-operation is essential to the highest success of a well-administered institution like a hospital or training school. There the workers have to give much of themselves in the discharge of their duties each day, and the call upon them for so large an output of sympathy, creates a want in many natures of sympathy for themselves and their work. So we have always favoured the encouragement of societies amongst the nursing staff of every large hospital, because in this way all who have received their training in such an establishment, whether they continue to work within its walls or are called to other spheres of labour outside, may keep in touch with each other, and by co-operation give and receive an amount of sympathy and encouragement which must otherwise be unattainable. Every matron of a large hospital, if her work is really to tell, must have large sympathies, and the gift to husband the resources of her staff so as to secure smoothness in working and the maximum of happiness and health for every worker under her control.

It has been the practice of the Nightingale School to have an "at home" once a year at the hospital, and to make it the occasion, not only of bringing the workers together, but of helping them, by counsel and opportunity, to make arrangements to keep themselves efficient, in many branches of the nursing field, with which they might otherwise find it difficult to keep in touch. We incline to the feeling that it would be well to make arrangements, as some hospitals already do, with each of the Nurses' Societies, to have quarterly or monthly meetings at the parent hospital, and to make such meetings the occasion for post-graduate instruction and the renewal of sympathy amongst all the workers connected with each school. After all it is essential that the principal of a training school shall promote in every way the greatest good of the greatest number. No doubt, in view of the multifarious duties which devolve upon the matrons, it is an act of self-denial to keep this principle ever in mind. Now Holy Week is a fitting time for everybody in responsible charge of a great palace of pain, as well as for every worker connected with it, to take stock of the past and its results, and to make special efforts to improve and strengthen every part of the organisation, which may tend to husband and enlarge the sympathy, which ought to exist, amongst all workers, who are here associated together in the ordinary course of the year's work.

Of course it may be felt by some that nursing is a business, and that a nurse who discharges her duties and receives her pay, need not worry about the higher principles which Holy Week brings home to the thoughtful. We would remind those, who have this feeling, that they cannot make efficient nurses, nor can they do full justice to themselves and their work, unless they recognise the privilege of personal service in the days of health in the cause of the sick. Sick people are dependent upon others, and especially upon the nurse, and unless there is a loyal determination to do the utmost possible by sympathy to soften in every way the trials of the patient, the attendant on the sick must be untrue to her higher nature, and unworthy of the best of which she is capable. The attraction which nursing has always possessed for women is probably largely due to this feeling. The best nurse is undoubtedly the best of women. Such a nurse never puts herself first, nor is she conscious in her work of her individuality, for the call of duty absorbs the whole of her energies which she so devotes to the fight with disease. A good nurse's first consideration is to secure the utmost happiness and comfort for her patient. Holy Week may then properly mark for all of us the enforcement of personal discipline, and the awakening of higher ideals made fruitful in better work.

### The Care and Nursing of the Insane.

By Percy J. Baily, M.B., C.M.Edin., Medical Superintendent of Hanwell Asylum.

### I.—ANATOMY AND PHYSIOLOGY.

(*Continued from Vol. XXXIX., page 378.*)

### The Circulation of the Blood.

The circulation of the blood is the function of the whole circulatory apparatus. We must, therefore, now inquire into the various uses of these different component parts which we have been studying. It will be convenient, at the same time, to describe the remaining portions of the vascular system—namely, the lymph and the lympathic vessels and glands.

*The Functions of the Blood.*—We have seen already that the blood is the medium through which the wants of the tissues are brought to them and the refuse matters which they no longer require are borne away from them. We may, in fact, say that the blood is the general carrier of the body. The circulatory system is like a railway system whose trains are continually travelling, taking up and setting down produce or passengers at every station. In like manner the blood is continually travelling through the blood-vessels, giving as it passes something to the tissues, and in return receiving something from them; and since this is the case, it follows that certain changes must occur in the composition of the blood during its passage through the blood-vessels. Now it is in the capillaries that the business of the blood is transacted and that these changes in its composition take place, and the function of the arteries may be said to be merely to convey the blood to the capillaries, while that of the veins is conversely to convey it back from them to the heart, the latter organ being the motive force which by its contractions sets the blood in motion, forcing it through the arteries to the capillaries, and from these through the veins back again to itself. As we shall see presently, when we come to trace the course which the blood follows, there are in our bodies two sets of capillaries; one set we will call the pulmonary capillaries—these are in the lungs—the other set are the general or systemic capillaries, which, as we already know, form a dense network of minute tubes in every tissue and organ of the body (except cartilage).

The changes which the blood undergoes in its passage through the systemic capillaries are different from those which occur during its passage through the pulmonary capillaries. The blood which is carried by the arteries to the systemic capillaries contains a relatively large amount of oxygen gas, and is also loaded with the nourishment which has been poured into the blood-stream from the walls of the stomach and intestines. Both of these (oxygen gas and nourishment or food) it largely (not entirely) loses during its passage through the systemic capillaries, and in exchange for these it gathers up from the tissues carbonic acid gas and various other waste matters or refuse. At the same time (that is, during its passage through the systemic capillaries) it undergoes a remarkable change in its appearance. The pure blood, which is contained in the systemic arteries, is of a bright red colour—arterial blood, as we may call it—but while passing through the systemic capillaries it loses this bright colour and becomes a dull purplish or even quite purple colour; it is then called venous blood. This change in its appearance is due to the diminished amount of oxygen which it contains. The artery which carries the blood from the heart to the lungs (pulmonary artery) is loaded with this venous blood, which during its passage through the pulmonary capillaries again assumes a bright red colour, the reason for this second change in its appearance being that while in these (pulmonary) capillaries the blood takes in oxygen from the air and gets rid of carbonic acid gas.*

Thus we see that the blood carries oxygen from the lungs and nutriment (food) from the alimentary canal (stomach and intestines) to the tissues, while from them it carries carbonic acid to the lungs and other waste matters to the kidneys and skin. It is, in fact, always (while in the capillaries) losing something and always gaining something.

Its sources of gain are:—(1) The alimentary canal (nourishment); (2) the lungs (oxygen); (3) the tissues (various waste matters and heat).

Its sources of loss are:—(1) The lungs (carbonic acid, water, and heat); (2) the tissues (nourishment); (3) the kidneys and skin (various waste matters, water, and heat).

During the whole period of life there is an incessant stream of blood flowing from the heart through the arteries to the capillaries, and from these through the veins back to the heart. This stream of blood naturally differs in quality in different parts of the circulatory system. Thus the blood passing into the lungs through the pulmonary artery contains the largest amount of carbonic acid and the smallest amount of oxygen. It also contains much food material taken up by the veins of the alimentary canal, and it is devoid of much waste material which has been removed from the blood by the kidneys and skin. The blood returning from the lungs differs from that going to them merely by being richer in oxygen and poorer in carbonic acid.

But not only does the blood vary in quality in different parts of the body; it also varies in temperature. The hottest blood is that which comes from the muscles, especially when these are in action, and from some of the internal organs; while the coolest is that which returns from the lungs and the skin. By the mixture of this warmer and cooler blood the temperature of the body is regulated and distributed.

Briefly, the functions of the blood are as follows:—

1. The respiratory function, by which is meant the carrying of oxygen from the lungs to the tissues and of carbonic acid from the tissues to the lungs.

2. The nutritive function.—The carrying of nutriment from the alimentary canal to the tissues.

* The "respiratory function of the blood" will be better understood after reading the section which deals with respiration.

3. The cleansing function.—The carrying away of various waste matters (other than carbonic acid) and water from the tissues chiefly to the kidneys and skin.

4. The regulation and distribution of heat.

The changes which the blood undergoes in the various capillaries may be summarised as follows:—

1. In the pulmonary capillaries (lungs) the loss of carbonic acid, water, and heat and the gain of oxygen.

2. In the systemic capillaries generally the loss of oxygen and nutriment and water. The gain of carbonic acid and other waste matters and heat.

3. In the capillaries of the alimentary canal—chiefly the gain of nutriment.

4. In the capillaries of the kidneys and skin. The loss of waste matters (other than carbonic acid) and water, and in the skin also the loss of heat.

But we have seen that in no part of our bodies does the blood come into actual contact with the tissue elements, for it is contained within a system of closed tubes—the blood-vessels. How, then, does this interchange of material between the blood and the tissues come about? The walls of the capillaries are so thin that much of the fluid part of the blood (the plasma) filters through them and fills the minute interstices around the capillaries themselves and between the individual tissue elements, so that these are entirely surrounded and are bathed by fluid. Not only the plasma, but many of the colourless blood corpuscles also find their way out of the capillaries into these spaces. This fluid which thus escapes from the capillaries is called the lymph. It is a little more watery than the blood plasma, but otherwise precisely resembles that, and "may be defined as diluted blood minus the red corpuscles." Now this lymph acts as middleman between the tissues and the blood. It receives on the one hand from the blood oxygen gas and food, and on the other hand from the tissues carbonic acid and other waste matters, and to the blood it gives what it receives from the tissues, while it gives to them what it receives from the blood. But this lymph also continually circulates, and finds its way back again to the blood. It does so by means of a series of vessels which are called the lymphatic vessels. These lymphatic vessels convey the lymph into a main lymphatic tube (the thoracic duct) which lies beside the backbone in the abdomen and thorax, and empties its contents into one of the large veins in the neck; on its way to the thoracic duct, the lymph passes through several glands which are called lymphatic glands. We need not here inquire into the function of these glands.

It sometimes happens, generally in cases of heart or kidney disease, that more fluid exudes through the capillary walls than the lymphatics are able to remove. In these cases the tissues become water-logged and the part swells. Such a condition is known as dropsy.

(*To be continued.*)

### The Nurses' Clinic.

### ACUTE DYSENTERY.

Dysentery is mostly met with in England in its chronic form and recovery is very slow and trying.

The patient having got over the acute attack or attacks abroad returns home in the hope of completing a cure. He is still feeling ill and weak and suffering considerable pain, is very depressed and nervous about his health, and takes an almost morbid interest in all his symptoms. The most difficult part of the nurse's work, but I am sure one of the most important, is to induce the patient to take a more cheerful view of life in general and of his own condition in particular. Sympathy he certainly deserves, but he must not be encouraged to talk much about himself, and efforts should be made to interest him in some quiet pursuit which will occupy his time and thoughts without tiring him. The treatment and diet will, of course, depend on the doctor, but much tact and patience will be needed in regulating the latter. At one time the patient may persist in refusing nearly all food in his fear that it will bring on an attack of pain and dysentery, and at another he will be tempted to eat the very things he should avoid; so that with a strictly limited diet it is sometimes difficult to make him take a proper amount of nourishment. The stools should be inspected by the nurse every day, and when necessary kept for the doctor to see. She should watch for any signs of blood, mucus, undigested food, constipation, or diarrhœa. In the case of a very nervous patient it is a good plan to examine every stool, as very often a trace of blood or mucus which is due perhaps to piles or constipation is exaggerated by his fears into a return of hæmorrhage.

The frequency, too, of constipation between the attacks of acute dysentery is another reason for daily inspection. Some patients require an aperient every night, and the amount given must depend on the action of the bowels during the day. Ol. ricini is generally ordered, but sol. mag. sulph. may be given where oil is much disliked.

The oil should be given in coffee, brandy, or lemon syrup, but whichever is used the glass should be well warmed first, then pour in about ʒij. of hot water, ʒss. brandy or lemon syrup, and then the oil, adding very gently, so that it covers the oil, another ʒss. or ʒj. of brandy. If the glass and the water were hot the oil will be suspended without touching the sides of the glass, and can be taken without being tasted at all. A piece of lemon to suck will prevent the mouth feeling greasy afterwards, or some people prefer a piece of dry bread. If coffee is used it should be hot enough to act in the same way.

In an acute attack, which may be brought on by chills, improper food, worry, etc., the patient has intense griping pain which comes on in paroxysms and is agonising in character—the abdomen is tender and distended, the temperature is raised, the pulse quick, the face soon becomes pinched and sunken, the expression anxious. The stools, which may at first be constipated, rapidly become fluid and contain blood and mucus, until in a short time no fæces are passed at all and the motion consists of bright red blood and mucus only.

A starch and opium enema will probably be ordered to check the diarrhœa and relieve the pain. Hot flannels to the abdomen may do good, or hot fomentations with

### THE NURSES' CLINIC—*Continued.*

laudanum sprinkled on; but the latter are not so comfortable, as the great restlessness of the patient makes it impossible to keep them in place, and they are apt to make the bed-clothes damp.

If vomiting is present ice must be given to suck or sips of cold water to drink. The patient is, of course, much exhausted and as soon as possible some nourishment must be taken—this must be given cold and in very small quantities, ʒj. every fifteen minutes, increasing the quantity as quickly as it can be done with safety. Albumen water, peptonised milk, arrowroot, etc., may be given in this way. The mouth should be rinsed out frequently, but without worrying the patient, who must be kept as quiet as possible.

If the vomiting persists nutrient enemata must be tried; they must be given very slowly and carefully through a tube and funnel so as to avoid irritating the bowel, and should be discontinued as soon as possible.

While the bowels are acting constantly the patient's back will require great care and attention; it should be washed after each action and plenty of ointment—lanoline for choice—rubbed on to protect the skin and prevent it becoming irritated and sore. If the patient is much exhausted, it will be found better to put a large pad of wool under him, as the movement of raising him on to the bed-pan or slipper is very trying, and almost certain to increase the pain and tenesmus.

When the diarrhœa has ceased care must be taken that the patient does not become constipated. If the doctor has ordered the bowel to be washed out daily with warm water this will probably be sufficient to make them act, but if it is not the fact must be at once reported.

The washing out should be done with a tube and funnel, from two to four pints of water being used if the patient can stand it, but care must be taken not to exhaust him.

When he is convalescent he must avoid too much exercise or great exertion, and should wear warm clothes, a flannel binder coming well down over the lower part of the body must never be omitted.

The diet should be light and simple, and until he has reached his normal weight extra milk, cream, Benger's food, etc., should be taken between meals.

He should be weighed once a week to see what progress he is making.

### Incidents in a Nurse's Life.

### AN EPISODE IN A MINING DISTRICT.

Distances between the street lamps lengthened as I left the town farther behind me. I was on my way to visit a case on the outskirts of my scattered district, a small mining and manufacturing town not far from Manchester. There was no hospital within six miles of H—— and no nurse nearer than the next small town. So the boundaries of my district were vague, and my cases were many and various! On this November evening a drizzling rain fell; it was dark, and the unpaved road was muddy. Afar off I heard shrill voices, and saw moving figures under a gas-light. As I drew nearer I saw a lot of boys grouped round something that I could not see. Also I heard a loud gasping sound. I supposed that some dog or cat was being tortured for amusement and hurried to the rescue.

"What have you got there?" I asked, as I came up with the ring of boys. Seeing "tha' missus wi' tha' white strings" they made a gangway for me through the ring.

A boy, who looked about thirteen years of age, lay on the ground, breathing with loud, difficult gasps, that were almost sobs.

"How has he been hurt?" I asked.

A babel of voices began to relate some tale. I singled out one boy, and crying "Hush! hush! hush!" to the rest, told him to explain what had happened.

"Yon"—he pointed to the boy on the ground—"an a big chap fowt, an' yon licked t'other. Our Tommy"—here he pointed to a small boy who promptly ran away crying aloud—"ran and towd t'other lad's mother, an' she coom an' knocket our Mike down, an' hoult 'im, time her lad kiked 'is troat."

"How far off is his home?" I inquired.

"Happen, 'alf a mile."

On one side of the road was a disused mine and waste ground. On the other a high wall. About a hundred yards further on was light from a window. I bent down to look at the boy. His eyes were closed, and the face was white where it was not muddy. After I had unknotted his scarf and unbuttoned his shirt, I felt his pulse, and found it fairly strong and regular. My cloak was circular, and made of pilot cloth. I took it off, and, calling four boys, made them hold it, so folded that it made a practicable stretcher.

"Take his feet," I said to another boy; and, lifting his head and shoulders myself, we laid him on my cloak. I shuddered at the thought of all those dirty little hands, and the dirtier little patient! But the responsibility of life or death is urgent!

With six boys grasping the cloak and me steadying the head, we marched slowly to the dwelling where I saw the light. I knocked at the door, and a woman opened it.

"A boy has been hurt," I said. "Will you let him come in till I can get a doctor and have him removed?"

"Nay," and she shut the door.

"Go on," I said to my ambulance corps.

We went on for about twenty yards. Then a quick stride came up with us, and a man's voice asked:

"What's wrong?"

Thankfully I saw a tall young miner, whose good grey eyes peered down at us from a grimy handsome face. I told him.

"Where to art takkin' 'im?"

"To his home; about half a mile off."

He handed his bag to a boy with a brief "Bring it."

"Now, shape!" he said to the bearers, and, lifting Mike in his arms, settled him comfortably, with the boy's head resting on his shoulder.

"Tak' tha' cloak, missus," he said to me. "The'll be gettin' tha' death wi' nowt ower that cotton gown."

I shook the cloak thoroughly, and put it on. Anxiety had made me forget the cold and the rain. Then we started again. The boys ran in front, and I occasionally broke into a trot to keep up with the man's long, easy strides. Silence fell on us. I was considering the likeliest way to get immediate medical help. Our footsteps, and the boy's sobbing breath sounded strangely loud.

"Gin 'ee breathe that gate, ee'll not gain 'is whoam leevin'" the man remarked.

When the lights of the hamlet where the boy lived came in view, I sent two messengers to the nearest doctor, and told them, if he were out, to go to another.

"Anyhow," I said, "don't you come back without one."

"Reet, missus!" and off they scampered.

The boy's breathing changed suddenly. Either he was sinking, or the injury was not, as I had supposed, mainly to the throat. We reached a row of dwellings, and our guides pointed to the last of the row and shouted :

"Yon's t' house."

At the door the boys held back a little, and the miner, who was just in front of me, lifted the latch and walked in, carrying his burden. A man sat smoking by a great, glowing coal fire. A woman was peeling potatoes at a deal table. She looked towards us as we entered, gave one ear-splitting shriek, and rushed past us out into the road.

"She's skeart," the man observed, as he laid his pipe down in the fender, very carefully, and came towards us. "What's t' young fule gotten noo ? "

The miner laid the boy down on the sofa, then he answered :

"Aw canna' tell 'ee. Lad, gie me yon bag. Good neet t' ye missus."

"Thank you so much," I said. But before the words were spoken he had vanished into the night. Would the father vanish next ?

"Don't you go," I said nervously.

"Nay, aw'm bidin' t' whoam," he answered.

The boy began to move his head and mutter. The man was intelligent and handy. He gave me a "moog" of hot water, with soap, flannel, and towel, filled the kettle and put it on again, and found a quart stone bottle. Meantime neighbours crowded into the room. I had been a district nurse too long to ask them to go out. Besides making oneself disliked, such requests are generally useless. Only the doctor can clear a room—at least, during his visit! The comments of the crowd were frank and various, respecting both the patient and the nurse.

After I had put a pillow under the boy's head, and a blanket over him, I cut some hair away from a small bleeding place on the scalp, and bathed it and the ear. We use cotton from the mill in the cotton districts, and I always had a little packet of it in my apron pocket, even when, as now, I had not got my bag. While I washed his face and hands his father took off his clogs and socks. His feet were quite black.

"Hold the basin, please," I said, and with plenty of soap and bathing I did all I dare under the circumstances. When I had dried them they were pie-bald, and in some degree pervious to the heat from the bottle I put to them. The boy's movement and muttering had become mechanical and regular as clockwork. He moved his head from side to side, with four jerks each way, and with the first two jerks from right to left he said "Unto." For some moments I counted.

"Unto," three, four, five, six, seven, eight. "Unto," three, four, five, six, seven, eight.

He swallowed the first half-teaspoonful of milk I tried him with; the second he spat in my face, and afterwards clenched his teeth together. His temperature was 103.

"Isn't his mother coming back ? " I said to the man.

"Happen un may," he replied, without interest.

A woman in the crowd explained.

"Yon's a bad lad, oft foightin', an' browt whoam 'alt kilt. That drive 'is puir mother inter skrikin hysterics. I reckon un's in Theresa Mary's."

I sat down and waited for the doctor, and devoutly hoped he would not be long in coming. As I had washed the boy and had not given him brandy I knew what my crowd of critics would say if the boy died before the doctor came— "Sithee! yon missus done that!"

In about ten minutes I heard the doctor's cheery voice as he pushed his way through the crowd outside the door.

"What's the young rascal been at now?" he said as he came in. "Glad to see you here, nurse."

"Congestion of the brain," he declared, after a brief examination; "I suppose his mother's having hysterics at a neighbour's house? Lizzie Jane," he said, singling out one from amongst the women : "You go and tell her to come to my surgery at once for medicine for the boy, and advice for herself."

Lizzie Jane went. The doctor spoke to me so that all could hear :

"Get him undressed, nurse, and wash him as much as you like; and when his mother comes, go home; I'll tell her what to do; and that young scamp will be running about again in a day or two."

Then he turned his attention to the crowd.

"One of you women stay to help nurse. Yes, Ellen Julia, you'll do. The rest of you come along home."

He went, driving the crowd out before him, and I heard their loud, jovial talking and laughter dying away in the distance. We all loved him, and his word was law in all that district. In twenty minutes the boy was in his nightshirt, and as clean as one isolated scrub could make him. He had ceased to move his head or speak, and I noticed signs of returning consciousness.

Within half an hour his mother came in. She had a bottle of medicine in her hand, and looked rather shamefaced. She bent over the boy and asked : "Art mendin', thee little deevil ? "

The boy's eyes opened. He spoke feebly.

"Thee go ter h——, an' ne'er moind me! "

His mother stood up and laughed happily.

"'E's grand ! " she said. "Thankee, missus."

The man had long been sitting by the fire, smoking his pipe in peace. He took it out of his mouth, glanced at his son, and remarked :

"Aye ! thee'll mend. Nowt cooms ter nowt ! "

### Nursing at Sheffield in the Eighties.

In the face of all that it is thought—and rightly thought—necessary to do for the comfort and education of the nurse of to-day, it may be a matter of interest to recall for the benefit of the younger nurse-readers of this paper something of the disadvantages under which the nursing of one large English hospital was done in the early 'eighties. A short time back I read the story, "A Raw Probationer," and then I came across reviews of it in more than one professional journal condemning it as exaggerated—improbable even—in its portrayal of hospital life. To me, remembering my own beginning, it did not seem so. The Redburn Infirmary of the book and the Sheffield Infirmary of my earliest recollections have, indeed, many features in common. The matrons especially might have been twin sisters, the lady under whom I worked having been quite as autocratic, despotic, and inelegant as ever Miss Warner was, while the hours of duty—from 7 A.M. to 9 P.M.—were precisely similar in both institutions.

### Dining in the Bedrooms.

At Sheffield in those days there was no dining-room, even for dinner. The midday meal, like all the rest, was eaten in the bedrooms, which adjoined the wards. Two or three days a week it was cooked there, though on these occasions the nurse generally waited for her dinner until supper time, because in the middle of the day, if she had stopped to cook it she would have had no

### NURSING AT SHEFFIELD IN THE EIGHTIES—*continued.*

time to eat it. The Sheffield General Infirmary had then 160 or 170 beds—none of them ever sufficiently long unoccupied to get properly cold. Its staff of nurses was seventeen, including five night nurses and three probationers. Four night nurses only were on duty at one time, the fifth being the relieving nurse, so that each had one whole night off in five. They needed it; the night nurse of our "landing" had seventy patients to attend to, women and children, medical, surgical, and ophthalmic. How was it done? Nobody could say, but there were rarely complaints. Of the three probationers, or assistant nurses, as they were more commonly called, only two belonged to the infirmary proper, the third was merely in training for the nurses' home in Glossop Road, and was replaced every nine months —that period being thought long enough for a nurse to train for private work. The three were distributed amongst the principal surgical departments in the day time—twenty-four beds to one nurse-in-charge, a probationer, and a wardmaid. In medical wards of fifteen to eighteen beds, or in surgical wards of twelve patients only, the nurse, except for the wardmaid for scrubbing, washing dishes, etc., worked single-handed. The times off duty for the day nurses were one evening a week from 6 to 9.30, one afternoon from 2 to 9.30, and every alternate Sunday from 2 to 9.30; and in the case of the wards where there was no under nurse, then the nearest nurse did double duty for the time being.

### The Nurses' Rations.

There was no special time for meals; the head nurse stole a few minutes from her wards when she liked or found it convenient; the under-nurse when the head nurse gave her leave. On specially busy days, such as operation days in the surgical wards, or when admissions were heaviest or inopportune, a meal was not infrequently omitted altogether. The rations per head delivered in the nurses' bedrooms every Monday morning, and kept in a locker placed there for the purpose, consisted of ¼ lb. tea, 1 lb. sugar, ½ lb. butter, and sometimes ½ lb. cheese; but that was an extra not to be depended upon. These, with bread and milk, procured from the ward supplies from day to day, provided all that was thought necessary for the breakfasts, teas, and suppers for the week. But there was one other item, a bottle—large size—of beer or porter daily. No premium was offered to teetotalism. Occasionally a nurse who had views on this head asked to have cocoa substituted, but she was generally snubbed and told if she did not like what was given her she could go without. The only one who during my three years there did succeed in effecting the substitution of one small packet of cocoa for seven large bottles of beer a week had to buy her own extra sugar! So the nurses accepted the beer, and those who did not drink it found it come in usefully sometimes as a stimulus to convalescents to give more assistance about the wards.

### High Days and Holidays.

The only other dietary extra besides beer which the nurse enjoyed was one in common with the patients on such high days as Christmas, Easterday, and, perhaps, Whitsuntide. The probationer of each department where there was one—and the wardmaid where there was not—was summoned to the main kitchen in the basement, and so many little cobbles of very plain cake counted into her apron, one for each of her patients, and one each for herself and her head nurse. If there were patients in her department disallowed such luxuries she did not always feel it incumbent upon her to allude to the circumstance. She took all she could get, depending on the fairness of her head nurse to divide the surplus with her, or, where that prospect was not sufficiently reassuring, pocketed a cobble on her way back to the wards. But perhaps no greater test of the nurse's cheerfulness and endurance could have been devised than the daily dinners, consisting six days out of seven of beef or mutton and one vegetable, and brought to her room. Simultaneously with the wards' dinners it had, on the days when it was cooked, every chance of getting spoilt before she had time to go to it, by becoming cold if the room had no fire, or by drying up if there was one; and two or three days a week, as I have mentioned before, it was an uncooked meal which was served. I have no livelier recollection of the Sheffield General Infirmary than of the first occasion when, after a morning of hard work from seven to one o'clock with never a snack in between, I scurried to my room followed by an injunction not to be many minutes over my dinner, and found a raw chop and two raw potatoes reposing in the dish on my table! I always felt afterwards that I had successfully solved the meaning of "an all-gone sensation," alluded to sometimes in quack advertisements. Nothing but astonishment kept me from crying. "That's nothing" my head nurse said when, having fortified myself with a piece of bread and butter, I went back and told her. "I could have told you if I'd thought of it that it probably would be raw to-day as it was cooked yesterday and the day before; and then you could have been frying it in the ward kitchen while the ward dinners were being served. But never mind now, you can cook it for your supper, I always do that." "But what do they send it raw for?" I asked, not more than half consoled.

### The Reason for the Uncooked Meals.

"Oh, I don't know. I have heard it was on account of some nurses grumbling about the cooking at one time, and the matron hearing about it was so annoyed that she said they should all cook their own food for the future. Then some of the others complained that this was not fair—to serve everyone alike when only some had found fault and so there was a compromise—some dinners raw and some cooked." I have said that for six days of the week the dinners were the same, the exception was on Sundays when there was a sweet, and if any additional demonstration of the lack of inventive genius on the part of our matron were needed, that sweet surely furnished it. It knew only two changes in the twelve months, when it changed from plum dumpling to rhubarb tart in the spring and when it changed back from rhubarb tart to plum dumpling in the autumn.

### Anæmic Nurses.

Between the plain living and the high pressure at which we were kept going, I am afraid it would be no use pretending that we were not rather a peaky—not to say anæmic—looking lot, taking us all round, but we were wonderfully cheerful as a body, especially the three probationers. I question whether any three girls with so little reason for mirth ever found more to laugh at than they did during that one hour between nine and ten at night, when they met in their common room and compared experiences—often grim enough, goodness knows!—and cracked their little jokes over the spluttering frying pan before finally going to bed on a supper that ought to have been their dinner. There was no stated period of training, no certificate, no lectures, no technical teaching of any sort, but there was plenty of practice, for the students did no dressings, and we had books if we liked to give up our days off to studying them, which we very often did, so that one way and another I think that the Sheffield Infirmary licked us into very fair shape, and some of us, it may be hoped, did no subsequent discredit to our training school, rough and ready as we had found it.

### On Practical Nursing.

### FOR THE PROBATIONERS OF A CHILDREN'S HOSPITAL.

Nursing is a woman's profession, set aside for women, and a profession which in taking up we cannot be accused of usurping men's rights. It should, therefore, be our aim to make this profession, *par excellence*, what people are so fond of calling it, "the noblest profession of all." And every member ought to realise that she must do her share to uphold it and to raise the standard. It would indeed be an ideal profession if we all took up nursing for the true motive of this grand work—namely, the wish to alleviate suffering, and especially the suffering of the very poor. Few, however, start with this motive. We must all realise that the first impulse which made us enter the profession was one of the many impulses of the woman for seeking work: the necessity of earning money, dislike to governess teaching, restlessness, disappointment, antipathy to a step-mother or other unpleasant conditions at home, craving for excitement, etc., etc. But whatever reason may have made us take up the work, the moment we enter upon it let us all have but the one aim in view, and every one of us should look upon her work as a sacred trust. A nurse must not think that she is a martyr, but, on the contrary, realise that a great honour is conferred upon her in being allowed to carry on this most honourable profession. Unfortunately the public are too much inclined to uphold the idea that a nurse is a martyr and a saint. She may be a noble woman in the way she carries out her work, but she certainly is not a martyr. Every body of workers must have organisation. As it is quite inconceivable that an army should get on without discipline, so a nursing school, which is composed of so many different elements and has to fight an enemy quite as deadly as the soldiers fight, must conform to strict rule and discipline, and these rules must be obeyed without murmuring and arguing. As, again, it is important in a soldier always to present an appearance of neatness and cleanliness, so it is one of the important features of the outward appearance of the nursing staff; and this brings us to the vexed question of the hair, including the fringe. Though many a girl thinks very lightly of resigning the pleasures of society, dances, dinner parties, tennis, putting back the fringe is a heart-rending grief to her. As you will see later on, hair is one of the chief sources of dirt, therefore the less superfluous display of hair round the face the better. A nurse should avoid show; anything intended to attract notice being objectionable. The only thing a nurse should wish to attract notice by is the excellence of her work. Hence no jewellery or flowers are worn, and the uniform is made strictly according to rule. Aprons should be provided with buttons and not with pins, shoes should be made of plain leather. One of the greatest difficulties a new nurse finds is always to be up to time. If a new probationer trained herself to be methodical in the daily round of work from the beginning, always remembering before starting anything to have all the requisites at hand, unnecessary delay and muddle would be avoided. If, for example, you forget any of the articles needed when starting to wash a patient, this not only means loss of time, but often results in a chill to the patient which may have very serious results. Never start on a new task before completing one on which you are engaged. Do not skip your work because you are in a hurry, but rather go and tell the sister that you have not been able to finish in time, and then, instead of the work being left half completed, it will be done thoroughly. Everything must be cleansed and put back into the right place directly it is done with; strict tidiness is invaluable at any time, more so in one of the many rushes when we should be able to put our hand in the dark on the article required. Every part of your work must at all times bear the most minute inspection, every cupboard, the condition of the linen, the lockers, etc., etc.

### Loyalty.

As it is inconceivable to think of a soldier who is not loyal to his superior, so it is with a nurse. Many things may at first seem strange to her and she may be unable to understand the reason for them. But she must always bear in mind that those in authority have had a long experience, and that the rules and regulations of the place are the outcome of long years of practical work and thought. Criticism, discontent, and gossip must always be ruinous to harmonious working. A nurse who is able to appreciate the reason for the rules laid down may by her influence, used rightly, put a stop to all gossip and discontent and secure a right feeling among the staff, while often from a lack of moral courage, listening to grumbling, or, worse still, chiming in, she will help to destroy the harmony of the place. If a nurse, who has been some time in the hospital, finds the whole working of the establishment contrary to her ideas, she had far better for her own sake, and still more for the sake of the hospital, resign; a woman who cannot conform to rules and organisation will never make an ideal nurse, no matter how great her talents may be.

### Professional Manner.

Here, again, respect to seniors and superiors should be marked by deference and courtesy. A blunt and short manner when referring to a superior is most objectionable. A nurse should always rise when the doctor, matron, or sister enter the ward and remain standing during their visit, and should be ready to open the door for the matron or the visiting staff. Nurses should always inform the sisters or staff-nurse of the arrival of the doctor in the wards. A nurse should never diagnose. When required she should report clearly and concisely upon the symptoms she has been able to witness; but she should stop there. A nurse who realises her part of the work may be of invaluable service to the doctor and the patient. She may by careful watching and timely reporting save time, assist correct diagnosis, and thus facilitate a good result. We nurses are and never will be anything but the servants of the doctors, and good faithful servants we should be, happy in our dependence, which helps to accomplish great deeds. Think of the pride the nurse felt who was allowed to look after the first cases for whom the diphtheria antitoxin was used, or the nurse who was able to help Lord Lister in his grand work of introducing antiseptics. Surely, witnessing the beautiful cures in diseases which, before the discovery of these remedies, were almost invariably fatal, s reward enough. And even our everyday work gives us ample satisfaction. It is pleasant to hear that "this case of enteric could not have recovered without the excellent nursing"; to watch a dear, little patient who has struggled with death for days gently come back to us, and see that its first smile is for its anxious nurse. So let us all be eager, faithful, and humble servants to a noble cause and its priests on earth.

### Servants.

There is a great tendency in hospitals to undue familiarity with the servants. This familiarity ruins the servants, and makes the nurse's life very uncomfortable. At the same time the maids should be treated with kindness, civility, and consideration, and their work should be inter-

### ON PRACTICAL NURSING—*continued.*

fered with as little as possible; it must be remembered that through the carelessness of nurses in upsetting water over a newly-polished floor, etc., the work in which the servants have just completed has to be done over again; and for the servant to keep to time and to take pride in her work is just as important as for the nurse.

### Strict Honesty.

I am afraid that many of the nurses fail to grasp the full meaning of this term. We must understand that every penny which is given to the institution is given for the use of the sick, and that we, on the staff, are only considered by the public as instruments to carry out the administration of the money among the sick by means of nursing. This is one side of the question; another is, if we waste the supplies provided for the hospital more money has to be expended on those supplies than necessary, and it is distinctly a mild form of stealing to waste that which does not belong to us. Therefore exercise the strictest economy. This economy should also extend to the supply of food provided for the nurses : butter, jam, etc., should not be wasted at meals by leaving half of what one has taken. The meat should be carved as economically as possible. A very important point which is often ignored is that the ward supplies are provided for the patients only. A nurse thinks nothing of taking away for her own private use a bottle of glycerine, ointment, bandages, safety pins, pens, etc.; but if she would stop to think a minute before taking them away she would realise that they are not hers.

### Rules.

Every nurse must on entering a hospital endeavour to grasp thoroughly the meaning of each separate rule. The one most commonly ignored is the one about taking medicines on her own account; but nurses must understand that their rules absolutely forbid the taking of any medicine, not only from the hospital, but also from outside without the knowledge and permission of the matron. Any indisposition, however slight, must be reported, and then advice will be given and also the necessary supplies. It is strictly against this rule to take medicine on your own account or medicine advised by any one, except those in authority in the hospital, unless by special sanction of the matron.

### Habits of Health.

The first important item is absolute cleanliness, and a little detail which is very essential to remember in a hospital where the poor are nursed, is the guarding against the invasion of pediculi in the hair. While combing out a patient's head, a nurse must be very careful not to come in too close contact, and also train herself never to put her hands to her own hair. Each nurse should provide herself with a fine toothcomb and use it every night, and at the slightest sign confide in the sister of her ward. While speaking on this subject, it is well to again point out the advantage of doing away with the fringe. I should like next to impress upon you the necessity of rising early so as to acquire regular habits, and thus avoid taking frequent aperients. Constipation and the resulting indigestion, common complaints amongst nurses, are often induced by getting up at the last minute, thus leaving no time to attend to Nature's wants. There should be no rushing through meals, food to be digested must be eaten in peace. When off duty plenty of time should be allowed for getting back to the hospital without hurrying, as otherwise a rush at the last is almost inevitable, and it is impossible to begin work out of breath, if it is to be done properly. Nurses have a very unfortunate failing, that of drinking too much tea, and eating too many sweets and buns. The food provided by hospitals is quite sufficient for the requirements of the nurses—rich unwholesome food and constant drinking of tea only tend to upset the digestion and make nurses unfit for their work.

### Cuts on Fingers.

All cuts should be immediately reported, so that a dress ing can be applied. Nurses have a tendency to flat foot, swollen ankles, and varicose veins, brought on through constant standing. Flat foot can, to a certain extent, be guarded against by a very simple little daily exercise—namely, rising quickly on the toes and then falling back on the heels, as this tends to throw out the instep. This, though rather a tiring exercise, should be practised by those having a tendency to flat foot. Those with tendency to swollen ankles should raise the foot of the bed at night and further facilitate the onward flow of the blood by bandaging the feet and legs from below upwards. Nurses should go out in the fresh air every day if possible; and this is obligatory when nursing an infectious case. Nurses should regard their times off duty as real hours of relaxation, and throw off the cares and worries of the work as completely as possible. Most hospitals have access to tennis grounds and swimming baths. Parks and picture galleries are within easy reach of every London nurse. A ride on the electric trams or on the Thames penny steamers is most enjoyable, a stroll up Regent Street to view the latest fashions will attract others, so all tastes can be gratified. It revives you to have a real change, you come back to your work happy and fresh, and the mountains of care which seemed insurmountable before you started, have vanished—let us hope—in the blue sky.

### Nurses' Behaviour in the Wards.

A nurse should endeavour to be quiet, bright, and quick, and should never run, as running must create a draught, and cause an atmosphere of unrest which must be unpleasant to patients. A nurse must not hurry when administering to the wants of the patients, and should not, however tired she may be, let the patient feel that the work is an effort and performed because it is a duty. A nurse must possess great patience and sympathy, and this patience is required more in the nursing of children than any other branch of the work. An adult will take the medicine which is given him, but a little child must be coaxed to take it, and this will be easier, if we have patience to try and make it happy first, and so divert its attention from the nasty dose. A small child cannot say where the pain is, we must find out by careful watching. A child may be crying at the time when we want to take its respiration, and then we must wait. . . . But all this should not put us out of temper. Nothing is more depressing to a patient than cheerless surroundings, and here once more let us make the effort and be true to our colours. In our hospitals, where only the poorest of the poor are nursed, our sympathy and love should be given freely, fully, to the utmost extent. The time that is spent in our wards may be made the brightest spot in the little sufferers' lives, for it is too often the case that they come from a filthy home, where they have been accustomed to hear swearing, and have received nothing but rough usage since they came into the world. And if we try we may not only make their life while here pleasant for them, but have a lasting influence for good, which may help them in the temptations and struggles of after life. Let all of us who are in earnest, and have taken up this work in the true spirit, always remember that the alleviation of suffering does not mean bandaging wounds and giving medicines only, but primarily being in sympathy with the natures we are tending.

### Camps for Consumptives.

BY A NEW ZEALAND NURSE.

In the course of her work, a district nurse in Christchurch was much puzzled as to the best way of dealing with the consumptives under her care. She felt that the lives of her patients were of as much importance as those of people in better circumstances, and that as they must live somewhere, why not under canvas rather than in the cottages where others were open to infection?

The rates of the City allow for the feeding, housing, and medical attendance of the "sick poor," and the idea suggested itself to her that the difficulty of maintenance could be easily got over under these circumstances. Therefore it only remained to find tents, a suitable ground, and to remove the patients from their homes as soon as possible.

In December 1903 a plot of sandy land near the sea was borrowed for an indefinite time, four tents 8 by 10, with "flies" 12 by 14, and poles were given by a few sympathising friends, a space was cleared, and the tents pitched facing the north-west—the warmest aspect.

As the ground was overgrown with broom to the height of 12 to 14 feet, a good shelter was afforded against driving wind and rain.

Three men were placed there, the wife of one of them undertaking to live in another tent and look after them. During the day they lived entirely in the open air, and at night slept with the front of the tent wide open.

Each man brought his own bedding and furniture, and the nurse obtained a small camp oven, very similar to a gipsy cauldron, and as there was plenty of firewood about they cooked their own food.

The men, being in destitute circumstances, were supplied by the Charitable Aid Board with rations and medical comforts, such as milk, meat, rice, and other necessaries of life. In addition one man had a gun and so used to provide rabbits. The patients also caught fish occasionally, which made a welcome change in the diet.

In a very short time the enforced life in the open air showed good results, the weight and strength increasing. Once a week they went to see a medical man who kindly offered his advice.

As more patients applied for admission more tents had to be procured, and the management of the cooking and meals entirely out of doors became difficult. Some old timber and iron were given by a second-hand dealer; a builder gave some bricks; a door, a window, a large Colonial oven were also given, and soon a rough kitchen was put up by some of the patients, with a little help from friends.

Then a marquee 12 by 12 was bought with money collected, and one energetic lady begged some chairs, a table, and odd bits of furniture and crockery from different people, and in a short time quite a cheerful home was made.

For three months the camp lived in this hand-to-mouth condition. As it became better known, more gifts were sent in, and after a visit from the editor of one of the principal newspapers, contributions in money and kind were frequently sent.

As winter approached the nurse and her assistants found that the mist from a river near the camp affected the patients. Moreover, the residents of the neighbourhood were agitating to get the camp removed; so the nurse set to work to try and buy a piece of land to prevent the patients being constantly "moved on."

After looking at various sites, an old patient, who had a fowl-farm on the sand hills, offered his land, consisting of nine acres, with a small cottage, for £250. He had lived there during a winter and was much benefited in health, so his offer was gladly accepted. Money continued to come in, which enabled the nurse to buy the land and move her patients on to it.

About that time she began to need help in the administration and formed a committee of working men to help carry on the work and collect funds.

A flagstaff and flag were given by some wharf labourers to mark the position of the camp. The nurse divided the tents into groups, and formed three small camps of seven or eight tents in each, presided over by a matron, whose cottage was in the centre and close to the marquee and kitchen. The marquee was erected on a frame and a fly sufficiently large to form a verandah on either side.

During the winter the patients stood the cold well. One or two gales wrecked some of the tents, but high breakwinds which formed a good shelter were built. Demands for beds increased, and friends and sympathisers gave tents and shelters.

Inside a Tent in the Camps for Consumptives.

The shelters are made of "malthoid," 8 by 8, three walls 8 feet high, the front side being fitted with a blind drawn up by the patient when in bed. The roof is in the shape of a Swiss chalêt, which is raised sufficiently high to admit of ventilation all round, and there is a ceiling of buttercloth muslin (see picture). The shelters are fixed at the four corners by pegs driven some feet into the ground.

One shelter was paid for with money collected in pennies from the boys of a public school. Various societies gave shelters and tents, and now the patients number twenty-three. The staff consists of a trained nurse as matron and a man cook. The patients, being more or less in a curable condition, are able to do most of the routine work of the camp. This has been found of benefit to them, and is there-

### CAMPS FOR CONSUMPTIVES—*continued.*

fore made part of the treatment, the work for each man being allotted weekly by the medical man. The honorary physician has a private sanatorium close by and visits the camp at regular intervals.

Very strict rules are made for the disposal of the sputum. Each patient is provided with a sputum flask, which is carried in the pocket, and any patient found expectorating on the ground is liable to be expelled. A rough furnace, where the sputum is burnt every day under supervision, has been made, and the flasks are boiled.

Outside a Tent in the Camps for Consumptives.

Some time after the men's camp had been launched, the attention of the originator was drawn to the necessity for something being done for the consumptive women. She called a public meeting of women only, which was well attended, and addressed them upon the subject of this disease, and appealed to them to do something for their fellow-sisters. The response was good. The first offer she had was the loan of four acres of land with a house, some two miles from the men's camps, well sheltered and on a sandy soil. Tents, shelters, and furniture were given by various women's unions and societies, and many others contributed subscriptions and household goods. A committee of ladies was formed, and now there is a camp in full working order capable of treating sixteen women.

Then a difficulty arose as to a means of protection for the women at night, as the camp was open to the surrounding country.

A fence 8 feet high was put up with rows of barbed wire about 6 inches apart, the top slanting inwards to prevent anyone getting in or out. This form of protection was found very satisfactory, as it does not exclude air or outlook and gives the patients a feeling of security. The staff for these patients consists of a trained nurse, a sub-matron, and a man to do the rough work.

The women's shelters are in three rows, and each shelter has an electric bell communicating with the matron's bedroom, in case of sudden illness at night. There is a routine of treatment set by the physician similar to that in the men's camp, special attention being given to breathing exercises. Care has been taken to admit only those in the curable stages of the disease, as advanced cases have a bad effect on the others, and would require a larger staff. The life is very monotonous, as the cure is slow, but a piano, books, and games help to pass the time, and sometimes friends from town give concerts and entertainments.

The number of cures averages much the same as in other sanatoriums, and the founder is sure that the more simple and primitive the life the better. Experience has shown her that tents are preferable to the shelters, as the wind can blow right through the canvas and at the same time the tents are warm. Many patients share this view. In warm weather the sides of the tents are rolled up. As good results are obtained in winter as in summer. The spring winds are apt to cause pleurisy, especially amongst the men, as they are more careless, and as a safeguard blue worsted guernseys have been provided.

The average cost per head is 10s. per week. A charge is made when the patient can pay; the amount is determined according to the means of the patient. Anyone unable to pay for treatment in a private sanatorium comes under the heading of "poor," and is therefore eligible for admission.

### What Nurses Owe to the Medical Profession.

BY A FORMER MATRON.

Perhaps very few of us as nurses realise the deep obligations we are under to the medical profession from the very beginning to the end of our nursing career. As would-be-probationers, one of the first steps we take is to ask our family practitioner to fill up a medical certificate which shall guarantee that our state of health is sound enough to warrant our introduction to the arduous duties of a hospital ward. Then when all the initial formalities have been successfully carried through and we find ourselves really established as nurse-probationers in a larger or smaller hospital, as the case may be, how much we may learn daily from the medical staff if our ears are quick and our eyes are open to observe what is passing around us! From the hospital surgeons we learn the use of instruments and surgical appliances, and the meaning of aseptic surgery. They also give us object-lessons in the application of remedies and practical demonstration of the art of bandaging. From the physicians we learn, perhaps unconsciously, the value of objective symptoms in the treatment of disease, more or less of the effect of different medicines, and the significance of posture in various illnesses. Very much knowledge may be acquired from daily observation by the alert nurse who wishes and means to become an intelligent assistant to the doctors under whom she may work in the future.

Then we are under great obligations to the members of the hospital medical staff who lecture to us on anatomy and physiology, etc., for somehow we get more light on a subject from a lecture than we do from private study of the very excellent text-books lent us by the matron. We older nurses look back with gratitude to the kindly interest evinced by the staff in our progress, from one position to another in the wards, and remember how they were always ready to enlighten our ignorance and explain knotty and puzzling points to us, as we felt that the all-round nurse needs to know something of the theory as well as the practice of nursing. But doctors befriend us in other ways. Besides teaching us the fundamental knowledge of our profession, they invariably support the nurse in the event of any patient disputing her authority in the wards, although she may be told privately that she has been rather unwise; and are always ready to put the best construction on failings of judgment or misdirected energy; though mistakes in hospital work are always more or less serious.

When we have finished our three or four years' course of training, we usually seek testimonials from some members of the hospital staff to help us in gaining a suitable post in one of the many branches of the profession open to nurses, and such testimonials are usually very cheerfully given. Should our tastes lie in the direction of private nursing, either connected with institutions or on our own account, then we depend entirely upon the medical men of the neighbourhood we elect to settle in, for our living, and, as a rule, they will support a nurse who loyally obeys instructions and is thoroughly reliable. All the way along the medical profession are our best friends, and right feeling and gratitude, not to mention the lower motives of policy and interest, should make us absolutely loyal to them, so that we may never by word or look do anything to lessen the confidence of a patient in his or her medical adviser.

So far I have said nothing about our obligations to doctors in times of personal illness. Then, indeed, we are treated as colleagues, and receive the most kindly attention, and are the objects of the highest consideration, entirely without question of pecuniary recompense. Not only in England, but even on the Continent, nurses are treated in the same generous fashion; the best skill is at our service, and no effort is spared to restore us to health and make us fit for work again. Such considerate treatment demands of us the highest return we can make, which is intelligent assistance and unswerving loyalty. The confidence reposed in a good nurse by the medical man whose patient she may be nursing, makes her work a pleasure and causes her to lose sight of the petty vexations and small worries which inevitably accompany the duties of a nurse, either in the wards of a hospital or in any other department of nursing. One of the strongest reasons why trained nurses are so universally employed in these days in cases of serious illness is, because a medical man feels that he can rely upon a woman who has gone through the discipline of a hospital training, and he knows that his instructions will be faithfully carried out, whereas the friends of the patient are prone to humour him and quietly ignore the doctor's orders.

Personally, the writer can only express the profoundest gratitude for assiduous medical attention, coupled with the most delicate consideration during a long and trying illness, besides which she gladly acknowledges the skill and kindness of one of London's most eminent specialists in the performance of a very critical operation to the success of which she knows she owes her life. Undoubtedly there are hundreds of nurses who can speak in similar terms of kindnesses received in like circumstances from the medical profession, and as the subject is not very often publicly referred to, perhaps this paper will serve the double purpose of reminding nurses generally of the debt they owe to the medical men of England, and at the same time show that nurses are not ungrateful for all the benefits they receive, although they may not often openly acknowledge their indebtedness.

### A Medical Mission Hospital in India.

A new nursing superintendent's first impressions on going into a mission hospital for native women and children in India are not often favourable, I fancy; compared with the picture in my mind's eye of the long, clean, and tidy wards of an English hospital, I, at least, was not much impressed with the first view I had of Indian ones. The air of spotless cleanliness, neatness, and order which is so apparent to even the casual visitor to one of our home hospitals is chiefly conspicuous by its absence abroad. The beds, for instance, almost make one's heart ache; it seems so impossible ever to make or keep them even clean, not to speak of tidy.

### Indian Beds.

A wooden frame laced across with plaited string is what the Indian woman loves best to lie upon; this, with a cotton mat, a folded blanket, and a thin sheet upon it, is usually all she requires for bedding. I used one of these bedsteads myself for some time, and found it very comfortable, but then I, of course, had a thin cotton mattress upon it. One advantage about them for hospital beds is that the string can easily be renewed when dirty or worn; also they are very inexpensive, and light enough for four nurses to carry with the patient on out into the couryard. When the sun has set in the evening one sees nearly all the beds out in the courtyard inside the hospital buildings, so that the patients can enjoy whatever coolness there may be after the heat of the day.

### Sterilising the Beds.

When I arrived at my first hospital in the North of India a novel way of making the beds free from inhabitants other than patients had just been invented—namely, by boiling them. This is sterilising carried to further lengths than we in England have yet reached; but it was necessary to get the bed thoroughly clean or else to throw it away, so dirty do the beds used by the natives become, in spite of all care possible. So a huge boiler made very inexpensively out of, I think, kerosene oil cans, had been constructed, large enough to cover half the bedstead at one time standing on end in the water. Under the boiler was a space for a charcoal fire, and each half of the bed was well boiled for five or ten minutes, and the result proved most satisfactory. Now the process is repeated at intervals whenever necessary.

### The Patients' Friends.

Another reason for the untidiness of the Indian wards is that nearly every patient, when admitted, brings a friend in with her, as both women and children are too timid as a rule to come in alone among strangers. It has been found necessary to allow this practice; but the number is strictly limited to one attendant; the latter also gives reports to the friends at home as to the patient's progress, and sees that nothing that can be helped is done to break caste while in the hospital. Of course, the very fact of their coming into the hospital at all in many cases breaks their caste, and for this they have to go through certain ceremonies on their returning home, in order to become reinstated. The companions who come with the patients are not provided with beds, and either sit or lie about the wards during the day, and sleep on a mat by their friend's sides at night—not very pleasant for the night nurse. Then, too, they bring their own cooking utensils, usually made of brass, with them, and these, though intended to be kept in

### A MEDICAL MISSION HOSPITAL IN INDIA—*continued.*

the lockers by the beds, are often left scattered about the floor, and it does not do to be too strict in our efforts at tidiness abroad or our patients simply would not stay. However, when we English nurses become a little more used to things, and learn how very different the patients are in the hospital from what they are in their own homes, we become thankful for a fair amount of order, and really begin to wonder why things seemed so terrible at first.

### The Difficulty of the Language.

The difficulty of the language is a very real one. In the first hospital where I was most of the nurses and medical students spoke English, and this was a great comfort. But in the next I went to not one of the nurses could speak a word of English, and I found it very difficult to direct them in their work, and was often at a loss for words in which to reprove them for things done amiss. I am afraid, too, I made very dreadful mistakes in my interpretation of their wants. However, one soon picks up the words in everyday use, and manages to get along as best one can; the people are wonderfully clever in understanding the new "Sister Miss Sahib's" way of trying to speak their language, and never think of laughing at her mistakes, however funny they may be.

### Indian Girls as Nurses.

I think the educated Indian girls, with good training, make very good nurses; but the difficulty is to get these from the upper classes of the people. In most mission hospitals the matrons have to be content with girls sent straight from orphanages, where they have been brought up. When the heads of these schools find a girl too dull, and not well enough up in her studies to become a teacher, and no one has come forward to marry her, they think that she will do "very well for a nurse"; so at the ages of from 16 to 18 the dull girls are sent to the missions for training, and with no real interest in their work, and still less capability for it, is it any wonder that they find nursing irksome work, and that we find it impossible to make them into good, efficient nurses? It seems a great pity that Europeans in India should consider girls of such a low standard of intelligence and education fit for the noble and difficult profession of nursing; and nursing superintendents long to raise the standard, so that their Indian girl probationers may not only do them credit after training, but may prove really useful nurses, fit for any post they may have to fill in the future. When the nurses come from the upper and educated classes, they mostly turn out really smart, clever nurses, and these are the kind wanted and sought for in our missions hospitals.

### Training Probationers.

It is difficult to teach our Indian new probationers to be aseptically clean in their work, and to keep the wards as they should be. They are not required to do nearly so much of the rough work as English nurses have to do in their first year of training, but they consider it beneath them in a land where no one but the sweepers, low-caste people, perform such duties. Then the sister has to show them by practical demonstration that she is not above such work, done for the good of her patients, and then they usually very soon become willing to do their share.

### The Patients Themselves.

With regard to the patients themselves, I did not find the diffculties so great as I had expected; the caste prejudice while they are in hospital seems to be much less than it was formerly, and as a rule now patients will take their medicine in a liquid form from the nurses, although it has been made with the dispensary water, whereas their drinking water they always take in their own brass or earthen vessels. As regards food, they have in most hospitals a Brahman high-caste woman as cook. She cooks and takes their food round to the patients at stated times, so that it may not be defiled by any low-caste person or Christian nurse touching it, a nurse, of course, going round with the cook to see that each patient gets the right kind of food. The daily hospital routine is as far as possible carried out in the same way as in hospitals at home, and each nurse has so many patients under her own special charge. The day begins with a short service, conducted usually by one of the lady doctors. No man is allowed to enter a Purdah hospital in India. All patients well enough to go into the ward in which the service is being held are expected to do so, but no one is compelled to attend. The singing, however, usually attracts them, as they are very fond of music, though their own is of the most primitive kind.

### Short Time in Hospital.

Operation cases, especially major ones, do very well in India. As a rule the women will not come in unless they can be operated upon immediately, and they often want to go out long before they are well. They expect to be cured very quickly. It is one of an English sister's chief trials that patients insist on going out long before they are ready to do so, and their excuses for going are sometimes very funny. Often a patient will forget and ask for leave to go home and bury some imaginary relative twice over, when the farce becomes easily apparent. A husband will come saying he must have his wife home to cook his food, and if reasoned with and told that his wife's cure depends on her remaining in the hospital, he will prefer her returning with him, even if she should lose her life through it, rather than put up with further inconvenience to himself. It is a great disgrace for a relation to die in hospital and "on a bed," so if a patient is not expected to recover, her friends must be told, so that they may take her home to die.

### Necessity for Economy.

In conclusion, I think one of the greatest difficulties of work in mission hospitals is the strict economy one has to observe in every single thing. The expenses are great, and the funds as a rule low, so we are bound to economise in every way we can. What would an English nurse think of only being able to put one thickness of antiseptic gauze, and that as small a piece as possible, over a wound because there were so few packets left, and it is so expensive, and so on with everything. The country-grown non-absorbent wool is used as much as possible for packing dressings, but it is not satisfactory. One feels after one has worked in a mission hospital more than ever before how great is the need of medical missions, and what a real help they may be, and are made, to the ordinary mission work, as healing for the body often leads to the patients and their friends becoming willing to learn of the great Physician, who loves our Indian sisters just as much as our more favoured English ones.

### To Nurses.

**We invite contributions from any of our readers, and shall be glad to pay for "Notes on News from the Nursing World," "Incidents in a Nurse's Life," or for articles describing nursing experiences at home or abroad dealing with any nursing question from an original point of view, according to length. The minimum payment is 5s. Contributions on topical subjects are specially welcome. Notices of appointments, letters, entertainments, presentations, and deaths are not paid for, but we are always glad to receive them. All rejected manuscripts are returned in due course, and all payments for manuscripts used are made as early as possible after the beginning of each quarter.**

### The Training of Cottage Nurses.

### A PIONEER ORGANISATION.

The appeal on behalf of the Cottage Benefit Nursing Association for financial help from the public, which was made for the first time the other day, suggested a personal inquiry into the origin of the movement, its objects, its development, and the system of training and instruction which is carried on under its auspices. The date on which it was started was January, 1883, and the title then, and for some years subsequently, was the Holt-Ockley Nursing Benefit Association. The founder was Miss Bertha M. Broadwood, and the work was limited at the outset to fifteen parishes around Ockley. Miss Broadwood, after much anxious consideration, came to the conclusion that the supply of nurses to country cottagers was one of the needs of the day, that they must themselves belong to the working classes, and that a small fee should in every instance be charged for their services. She also satisfied herself that these nurses could better put the theories learnt in lectures into practice in the homes of the poor in the slums than in the wards of hospitals with up-to-date appliances at their command. For ten years she acted entirely on her own initiative, and was the sole director of the organisation. In 1889 she secured admission for pupil monthly nurses—who have since been a great feature of the association—to lectures at the City of London Lying-in Hospital, and less than a year later she had the gratification of seeing her scheme for the special training in the elements of general nursing and maternity in district nursing adopted by Sister Katherine Twining at Plaistow.

### A Registry of Cottage Nurses.

From October in 1893 she commenced a continuous supply of pupils to Plaistow and other training fields; and in the same month a Registry of Cottage Nurses was started under Mrs. William Digby. Miss Broadwood has, in fact, been always a strong believer in registration as a means of supplying an absolutely authentic and accurate record of training; and from October, 1890, to the present time the nurses secured through the office of the Cottage Benefit Nursing Association and a good many others have registered. The certificate which is handed to them, not after the four to six months' training which they receive in one of the homes, but at the end of the three or four years, during which they are bound to serve some branch of the Association, describes their qualifications, the place of their training, their age, and home address, and is altogether so strictly a personal voucher of efficiency that it would be difficult for any one who desired to impersonate the possessor to succeed in the task. No nurse who does not satisfy the medical man and the Committee of the branch under which she has been working gets a certificate. At the end of 1890 Miss Broadwood, issuing the second edition of her pamphlet on "Nurses for Sick Country Folk," was in a position to furnish a list of fourteen existing benefit associations.

### The Start in London.

In 1894 the organisation had assumed such important dimensions that it was considered advisable to weld the associations affiliated to the central office together, and to establish headquarters in Buckingham Palace Road. Previous to this, in 1893, a meeting about benefit nursing associations was held at Lord Brassey's house. When the London office and registry was opened, and affiliation agreed upon, there were 56 associations in existence. In June, 1894, the first annual conference of the Affiliated Benefit Associations was held at 39 Berkley Square, and less than a year later the Association had increased to 65. In October, 1896, it was announced that this number had risen to 88.

### The Edmonton Home.

During 1898 the system of lending groups for the interchange of cottage nurses was set on foot, but it was not until May, 1900, when 130 associations had been formed, that the Training Home was opened at Bury House, Edmonton. As to the training which is carried on there, and the conditions under which it is given, it may be stated, as the result of a personal visit last week, that the institution is on a considerable scale. The house—which was formerly a country mansion, and still stands in grounds of its own with a wonderful tulip tree and a fairly spreading lawn at the back—is commodious and well situated for the purpose. There are dining and sitting-rooms for the nurses, and though separate bedrooms are not possible, their quarters are comfortable, the beds being curtained off. An ancient dairy has been converted into a spacious laundry, and the kitchen and outhouses are on an extensive scale.

### The Training and Instruction.

The Lady Superintendent of the Home, Miss Ellis, a fully-trained hospital nurse and midwife, and her assistants consist of a maternity sister and a district sister, who instructs in general nursing, both being also fully-trained hospital nurses. The theoretical instruction at Bury House, which is the model home of the Association, where the system of a regular sliding scale of fees for nursing all except necessitous cases was inaugurated, and now yields from £15 to £20 a month, consists of lectures on first aid by a medical man, while the Lady Superintendent and the district sister take the general nursing classes and the maternity sister the monthly nursing classes. But the principal point is the practical work, which is chiefly done in the homes of the patients. To help in their equipment, however, the probationers are taught plain cooking and housework, all having their household duties to perform, which are changed by the Lady Superintendent every fortnight and reported upon.

### The Class of Cases.

Practically every nurse goes in for a certificate of monthly nursing, which is put away at the registry until she has fulfilled her bond, extending over three or four years, as arranged. As far as possible, the probationers go out in couples, a senior and a junior. The district sister goes round with them, or follows them, and sees how the work of each is done day by day, just as she would supervise it in the hospital ward. The chief payments come from maternity patients, but the experience is very varied. Thus, in the report book, were cases of a nurse on night duty with a patient suffering from gout for eight nights; an enteric case ten nights; a pneumonia case four nights; a pneumonia case ten days resident; while there were records of a nurse in residence on a maternity case for two weeks, thirteen and sixteen days. There are no cases of free nursing save those recommended by medical men or the clergy, but the charge made is strictly according to the means of the patient, and the Lady Superintendent never refuses to send a nurse to an acute case. The number of probationers in the Home varies from seventeen to twenty-three. They receive their board and instruction gratuitously, and have only their laundry bill to pay. A candidate for admission must be at least twenty-three years of age. After the training the nurses receive a salary at the rate of £16 the first year and £18 the second, with a rise of £2 a year until £30 a year is paid. At the end of four years each nurse who has completed the period to the satisfaction of the branch Committees is paid an additional sum of £12. There are training homes in the suburbs of Glasgow and Bristol on the same lines as Bury House, and, like the Edmonton institution, they are nearly self-supporting.

The Latest New Departures.

In July last year it was determined to reorganise the central office, to transfer it to Denison House, Vauxhall Bridge Road, and to merge the affiliated associations into branches of the Cottage Benefit Nursing Association with a new constitution and a committee of management having a much enlarged staff of honorary officers. The constitution was drawn up by a representative commission, consisting of Dr. John C. Thresh, Officer of Health for Essex, Dr. Robert Boxall, the Countess of Ancaster, and Mr. Hall-Hall, the first three being now on the Committee of Management, with Lord Ancaster, Lady Bonham, Dr. Edward C. Seaton, and Sister Margaret, of 8 Durham Place, Chelsea. Having obtained their constitution, with elaborate and business-like rules, nearly 40 in number, the managers were not content until they got their own journal, and the first issue of a quarterly leaflet, called "The Cottage Nurse," made its appearance in November. The number of branches and associations in different parts of the Kingdom is now about 200, and upwards of 500 cottage nurses are employed; and the demand for suitable women between 24 and 40 is larger than the supply. Between March 1, 1905, and March 31, 1906, the number of monthly nursing pupils who received their certificate after examination by Mrs. Messenger at the Lying-in Hospital, York Road, London, was 45, and practically all of these had general training; while in 1905 the number of cases attended at Edmonton was 294. The offices of the Association are on the third floor at Denison House, and the business is divided into three departments—namely, the probationers, the registry, and the ordinary correspondence. Whatever criticism may be made as to the methods pursued credit must be given to Miss Broadwood for the energy, the skill, and the patience which she has manifested in building up a far-reaching system of cottage nursing on non-pauperising principles.

### Central Midwives Board.

The Central Midwives Board met on Thursday last week. There were present Dr. Champneys in the chair, Mr. Ward Cousins, Mr. Fordham, Mrs. Latter, Miss R. Paget, Sir William Sinclair, Miss Wilson, and Mr. Parker Young.

The Board accepted as read letters from the various bodies represented on the Board, appointing as members of the Board the same persons who have hitherto served in that capacity.

A letter was read from Dr. Meredith Young, Medical Officer of Health, Stockport, forwarding copy of a resolution passed at a meeting of the Incorporated Society of Medical Officers of Health on February 16, asking for information as to the proceedings of the Board so far as they affect Local Supervising Authorities. The Society especially asked for information regarding penal cases, the bodies authorised for giving instruction to midwives, the examinations which have been and are to be held, together with a list of places of examination, dates, number of examinees, and results of examinations. It was decided to send the information required to the Society.

The Secretary was instructed to reply to a letter from Dr. P. Ernest Nevins, of Liverpool, raising certain points as to the training of midwives, and the construction of rules relating thereto, to the effect that the Board had not laid down and did not intend to lay down rules specifying the exact routine of treatment in cases.

Examiners and their Remuneration.

The report of the meeting of examiners was then received. The meeting, at which Dr. Champneys was present at the request of the Board and also Miss Wilson, had been specially convened to consider the question of an official handbook for midwives. It was moved by Dr. Lyle and seconded by Dr. Fothergill that the examiners recommend to the Board the publication of an official handbook for midwives. After discussion, during which suggestions were made that the object of the motion might be met by amplification of the syllabus of instruction, the motion was put and lost, seven voting in favour of it and seventeen against.

Another matter discussed was the remuneration of examiners. It was moved by Mr. Wright and seconded by Dr. Stevens that the Board be requested to consider the question of additional remuneration for examiners travelling from distances. On discussion, the suggestion was made that the travelling expenses of provincial examiners attending meetings of examiners in London should be included in the scope of the motion. It was pointed out by the Chairman that, as no power to defray these expenses was given to the Board by the Midwives Act, objection would almost certainly be taken by the Privy Council to such a course being adopted. The original motion was then put and carried *nem. con.*

Mr. Parker Young held that the request of the examiners was for additional travelling expenses, and that it was within the Board's power to accede to it.

The question of making Cardiff an examination centre also came before the Committee. It was moved by Dr. MacLean and seconded by Dr. Swayne that the examiners recommend to the Board that Cardiff be recognised as one of the provincial examining centres. After discussion, during which attention was called to the bi-lingual difficulty among South Wales candidates and suggestions made as to the desirability of apointing a Welsh-speaking examiner for Cardiff or Bristol, the motion was put and carried by seven to five, the question of Cardiff being additional to, or alternate with, Bristol being left open.

A letter had also been received by the Board on the same subject from Dr. E. Walford, Medical Officer of Health for Cardiff, forwarding copy of a resolution passed by the Local Supervising Authority of Cardiff asking the Board to make Cardiff a centre. The matter was referred to the Standing Committee.

The Revision of Rules.

The report of the Standing Committee, which met on March 8, 15, and 22, was then taken. The chief business of the Committee was the revision of the rules, which were considered *seriatim* by the Board. It was, however, found quite impossible to deal with them all, and another meeting of the Board was fixed for April 10. Some important alterations of the rules were sanctioned by the Board. With regard to the fee for examination, the new rules admit a candidate to the examination who has previously been prevented from sitting through illness, for the fee of ten shillings and sixpence. On the motion of Miss Paget, seconded by Miss Wilson, the old rule that any candidate who had failed and again presented herself should only pay a fee of fifteen shillings was retained. It was argued that the failure was most frequently due to bad teaching.

Sir William Sinclair wished to introduce a rule requiring evidence of a certain standard of elementary education before candidates were received as intending examinees and entered on their training, his view being that the present rule, leaving rejection of a candidate on the ground of illiteracy in the hands of the examiner, was practically a dead letter. The matter was postponed until the next meeting of the Board.

It was decided to incorporate in the rules the rules of procedure in penal cases.

An important alteration was made in respect to the carrying of appliances to a case. The following clause was inserted: "The Local Supervising Authority may, in the case of untrained midwives, use its discretion with regard to insisting upon the carrying of a catheter and appliance for giving vaginal injections." Many cases had been reported to the Board of the improper use of such instruments by ignorant midwives.

The following important new rule was also added: "The midwife in charge must in all cases of labour examine the placenta and membranes before they are destroyed, and must satisfy herself that they are completely removed."

With regard to the regulation against laying out the dead, the new rules enact that a midwife shall not have transgressed in this respect if at the discretion of the Local Supervising Authority she (*a*) prepares for burial the body of a lying-in woman, a still-born child, or an infant dying within ten days; or (*b*) lays out a dead body in a case of non-infectious illness, provided that she is not attending a midwifery case at the time. In neither case must a midwife lay out the body of any patient on whom she has not been in attendance at the time of death.

Training Schools.

The Sheffield Union Hospital and the Parish of Nottingham Infirmary were approved as training schools.

It was decided that in future the lists of approved institutions and teachers be subject to annual revision, in the same manner as that of approved midwives, and that both approved teachers and approved midwives be required to furnish the number of pupils trained during each year.

The report of the Penal Cases Committee was received. A meeting of the Board was fixed for May 3 for the purpose of hearing six cases and any others that might then be ready.

### A Nightingale Nurse on the Pension Fund.

The writer of the following verses is one of the oldest of Nightingale nurses, having entered for training at St. Thomas's Hospital in 1861. She was eighty in January last, and has had an interesting experience, having assisted to move the hospital from St. Thomas's Street to the old Surrey Gardens, and from thence to the present hospital buildings on the Albert Embankment. She was at King's College Hospital for a few years, where she met Miss Agnes Jones, whose assistant she became on the latter accepting her life's work at Manchester. She was one of the first, if not the first, nurse to join the Royal National Pension Fund, having learnt the value of provident habits by being left at thirteen years of age, on losing both parents, to provide entirely for herself. This nurse is at the present time very well and strong, living happily on her pension and full of activity.

I wish that all good might befall every nurse,
That her spirit be light, but heavy her purse,
That she in her work be sincere and devoted
And for thought and sweet mercy for ever be noted.

That she'll ne'er feel the dread of dire poverty's pangs,
And this is the theme upon which my rhyme hangs,
Every nurse I'd advise to repair ere too late
To Finsbury Pavement; she'll find 28.

'Tis exactly in face of the Moorgate Street Station,
A large house it is where she'll gain information;
There in the office she'll be greeted with pleasure
And kind Mr. Dick will spend time without measure

In discussing the scheme and in hearing her views;
And I am sure she'll be glad when she hears the good news
That by joining this Fund she can save for a pension.
Then her mind will be free to work on without tension.

When her body's so tired that she's able no longer
To go on without strain, she can rest till she's stronger;
When too old for more work, independent she'll be—
The thought of it makes one both happy and free!

Of course you must practise a strict self-control
On pleasures and fancies, to win such a goal.
But your mind it would strengthen with each self-denial.
By joining this Fund to give it a trial.

When first it was founded, folk made such a rout,
And tried might and main to blow the light out.
But the match had caught fire and set it ablaze.
The foes, they were silenced, and looked on in amaze.

The Funds are so rich, they now count a million;
And I doubt not a year or two'll make it a billion.
I am one of the first who in it believed,
And for several years now have a pension received.

As the quarters come round, my annuity comes too,
From three policies funded!—the same you could do.
If it were not for this, why how poor I should be,
For I'm too old to work for my living, you see.

### Poor Law and the Central Midwives Board.

BY A CORRESPONDENT.

The organ of the Poor-law officers has published an article on the work of the Central Midwives' Board, which shows an insufficient grasp of the subject. The writer, in criticising the rules of the Board, overlooks a fact that was mentioned at the time, that these rules went before the Local Government Board three years ago and received various additions. The duty of the Central Midwives' Board is to see that the practical and theoretical training which they demand are sufficiently thorough to qualify a pupil to become a really efficient midwife. Any question beyond this is beside the mark. Although, as the writer of the article says, there are 11 Poor-law institutions recognised as approved schools compared to 37 voluntary hospitals, he omits to mention that a certain number of registered medical practitioners are recognised as approved teachers by the Board who, being workhouse medical officers under the Poor-law, can send up candidates for examination. The same plan of approving teachers applies to many small voluntary homes or hospitals which could scarcely take rank as training schools. If the contention of the writer is that all Poor-law institutions which have midwifery pupils should be simply approved without question by the Midwives' Board, the same principle would have to be universally applied.

It is curious that no one seems to realise a very important difference between Poor-law institutions and the smaller type of lying-in hospitals or homes; in the latter no case of general illness is admitted. In the large hospitals in which a system of teaching midwifery is now being revived after many years of disfavour, infinite precautions are taken to separate the maternity block and the staff from the general wards. It is obvious that only with such care can dangerous risks be avoided if maternity and general cases are under one roof. But it is a matter of fact that some Poor-law institutions have allowed pupils to take their cases while nursing in the general wards. Such a practice makes it incumbent on the Central Midwives Board to insist that the standard of separation between maternity and general cases shall be as complete as possible. In the older buildings this is not always easy, but much can be accomplished where goodwill and ingenuity are united with a quite small expenditure of money. The Board is perfectly competent to decide the number of cases essential to ensure good training; and Poor-law institutions, like others, must accept this ruling. It is acknowledged that the standard as regards cases was too low until the Board raised it a few years ago. The whole question of efficient training and the safety of the poor mothers who are compelled by destitution or by desertion to enter the lying-in wards of workhouses is far too important a one to be dealt with in any but the widest spirit, and the tone that animates the paper in question is therefore to be deprecated. There are two points that should be kept in mind in dealing with this subject: firstly, the difficulties that surround the work of a new Board which has on its roll a large proportion of untrained midwives; secondly, the need of dealing with the subject as a whole, and not as a part, relating to one set of institutions only.

### Practical Hints.

**We welcome notes on practical points from nurses.**

### THE CARE OF THE FEET.

It is not an uncommon sight, unfortunately, to see a nurse who walks badly, and this for the simple reason that the sound and perfect feet necessary for a perfect carriage are rarely to be found amongst those who have as much standing and walking as nurses. But, again, the fault rests not entirely in the feet themselves; the covering may be the cause and the effect an inability to walk without a tell-tale limp. The few hints that I can from experience give may prove a boon and blessing to some whose lives are at present far from enjoyable because of the everlasting pain and inconvenience of those poor feet.

In the first place, wear stockings of a rather thin texture without darns in the soles, of cashmere or something which is all wool. Then next in order are the shoes. If you wish to preserve the shape, and that means also if you wish to prevent flat-foot, do not attempt to wear the shoe of the one-strap slipper type with the so-called smart French heel; on the other hand, do not go to the other extreme, and wear the hideous concoction of flatness and squareness which some people would bribe you into believing "hygienic" ward shoes, for normal feet are neither flat nor square, so do not be led into placing your shapely foot into these dangerous and unnecessarily ugly shoes, for they are as harmful as their contrast. You must hit the happy medium, that is, a good, sensible walking shoe, preferably lace, so as to support the instep as well; choose those with neither pointed nor absolutely square toes and heels of medium height; having got your exact size without any pinching or unnecessary looseness, you have gained a great point. If it is suggested by some that walking shoes are too noisy for ward wear, rubber heels are by no means uncommon, and can be quite capable of rendering the shoes noiseless unless they "squeak," but that you must guard against when purchasing them. Here I will add a warning to the night nurse against the wearing of the flat felt slipper, especially if she has the slightest tendency to flat-foot. Leather shoes of some support should be worn, which can be made perfectly silent by adding a felt sole as well as the rubber heel if necessary.

Provided with the most sensible stockings and shoes procurable the start is fair, but supposing harm has already been done by too much running about in unsuitable foot gear, as prevention has not been allowed a chance, we must aim at a cure.

The habit of sleeping with the foot of the bed raised is wonderfully restful for tired feet. The bed may be raised easily by procuring two blocks of wood about six inches square, and hollowed at the top to prevent the legs of the bed from slipping off. The plan of alternate hot and cold bathing for the feet both night and morning is most beneficial, and after that a few minutes' massage will add to the comfort and efficacy of this treatment.

Also following this the sole of the foot may be well rubbed with boracic powder, also the soles of the stockings sprinkled with it.

A faithful following out of these few hints will, I am sure, do much to relieve and even cure the most weary and tender feet.

If there be a strong inclination to flat-foot you should, besides the foregoing treatment, also do your best to prevent the worst by exercises suitable and beneficial; the following may prove helpful:—

1. Standing on outer border of foot.
2. Tip-toe exercises; this may be also practised when going upstairs.
3. Standing alternately on toes and heels.

Whenever possible raise the feet when resting. At Gooch's Stores in Brompton Road can be bought supports which help to keep up the arch of the foot. Some nurses may say after reading this that these suggestions take up too much time to carry out, but surely it is worth while to spend a little time now instead of having to resort to the more severe treatment of rest in bed for some time, as must be the case in acute strain and flat-foot, so once again may I repeat that, if "Prevention is better than cure" the treatment is worth the trouble.

### Everybody's Opinion.

[Correspondence on all subjects is invited, but we cannot in any way be responsible for the opinions expressed by our correspondents. No communication can be entertained if the name and address of the correspondent are not given as a guarantee of good faith, but not necessarily for publication. All correspondents should write on one side of the paper only.]

### OPERATIONS ON MALE PATIENTS.

"A. B. C." writes from Newfoundland: I should like to hear through the columns of The Hospital whether it is usual for nurses to assist at such operations as amputation of the penis, dilatation of urethra and cystoscopic examination of male patients. Perhaps some of your readers connected with hospitals not having a medical school attached would tell me what is the custom in those cases.

### LINSEED-MEAL POULTICES.

"A Sister" writes: "A Matron" is right about making the linseed-meal poulties, with the exception that it should be so well beaten that it would not require either oil or gauze. Both in the fever and general hospitals where I was trained, the other way of putting the meal in first was considered most injurious.

### WAITRESSES ATTIRED AS NURSES.

"A Sister" writes: As a nurse of many years' standing, I write to complain of what I consider to be an indignity to the profession, and that is the way in which our uniforms are copied and travestied by waitresses. I am staying at Bournemouth, and on visiting the Bungalow Tea Rooms there this afternoon I felt annoyed to see that all the waitresses there were dressed exactly in the uniforms of "Sisters," with "Sister Dora" caps and strings. I venture to suggest in the interests of all nurses that the proprietor of the restaurant should be asked to desist from dressing up his servants in the respected uniform of the noble army of nurses, for there should be ingenuity enough to design some other dress instead of copying ours.

### DISTRICT NURSING AMONGST THE JEWISH COMMUNITY.

Mrs. Model, 105 Fellows Road, N.W., writes: The Home consecrated by the Jewish Rabbi is intended to accomodate four district nurses, probably two for general work and two for maternity work amongst the Jewish poor in Stepney, Whitechapel, and Spitalfields. The Sick Room Helps Society holds the place of a woman's sick benefit society as it enrolls members who, for a minimum payment of 10s. in weekly instalments, can obtain the services of a help—*i.e.* handy woman—to come in for two weeks daily, to wash mother and infant, cook, clean, and wash for family. Trained maternity nurses superintend this work, and, where required, their professional assistance is given. Cases are attended after delivery only, on presentation of certificate of doctor or of midwife. The services of sick nurses will be given to members, or free at doctor's request. The work will be conducted on the same lines as that of the East London Nursing Society.

### MONEY PRESENTATIONS.

"A Queen's Nurse" writes: Under the heading "Money Presentations," of March 31 you remark: "We are utterly opposed to money presentations as a matter of principle." I am very glad to hear the practice denounced. That nurses and other workers should receive an adequate remuneration for services rendered is, of course, right and proper, for "the servant is worthy of his hire"; but it is lowering the standard to accept what is beyond our due. Supposing an employer to be particularly pleased with the services rendered by one under him—he offers him or her a certain sum over and above his wages—if the person concerned feels her own dignity, she will refuse, and by so doing she will show that she performs her work for its own sake, not for the sake of a reward. The giving of a suitable memento on the departure of a colleague is a graceful act, provided all who contribute do so with a willing heart. It is not difficult to find out the tastes of people we have long been in contact with, and how much nicer to make a presentation in kind than it is to give a lump sum of money—it savours rather too much of the market or the bargain—as if we were paid off and dismissed.

### THE NURSES OF THE PARK HOSPITAL, HITHER GREEN.

"A Trained Nurse" writes: May I protest against some of the statements made to your Commissioner by the Matron of the Park Hospital, Hither Green, *re* the very little knowledge a hospital-trained nurse has of ward administration or the syringing of throat and ears until she goes to a hospital under the Metropolitan Asylums Board. I myself was trained at one of the largest general training schools for four years, afterwards going to an Asylums Board hospital to gain some practical knowledge of the different fevers, not to learn ward administration or throat and ear syringing. Those things I learnt during my training; and, having had entire charge of wards both in a general and an Asylums Board hospital, I can certainly say I found the wards of the latter much easier to manage. With regard to the clothing of patients while in an Asylums Board hospital—that, surely, does not make such an amount of difference to the management of the wards? All the London Poor-law infirmaries do the same thing, so that Asylums Board hospitals are not by any means unique in this respect. Then, as to the amount of care to be taken in disinfecting and cleaning generally, has the Matron of the Park Hospital never heard of a case of fever being discovered in the wards of a general hospital? And, in the event of such a thing happening, does not the question of thorough disinfection assume a much more important aspect under the circumstances? One would also gather from the Matron's remarks that wardmaids are an unknown luxury in the wards of a general hospital. I think that if she were better informed on this subject she would find that quite as much scrubbing and cleaning is done daily by the wardmaids in a general hospital as by those in fever hospitals, and are not fresh air and soap and water important factors in the treatment of all diseases?

### NIGHT NURSES' HOURS.

"A. B. C." writes: I have carefully read the letters in your recent issues, and to me it appears that the writers are simply stating facts. Why does "X. Y. Z." call them grumblers, and so very positively state that these correspondents are a "nuisance to their matron, sisters, and the poor patients"? Does the cap fit "X. Y. Z." so very nicely that she thinks it must be some of her own staff who write these letters? It is very evident that the members of her own staff whom she mentions have had serious illnesses, were in a very run-down state, or they would not have taken the diseases which "X. Y. " mentions. I cannot help wondering if they were not "overworked?" No doubt these nurses would find it awkward to accuse their matron of "want of sympathy and unkindness" when they were such good "cases." "X. Y. Z." quotes her own want of both in the case of headache and "small ailments." I can quite imagine that "X. Y. Z." is one of those who only get "serious illnesses" themselves. We have all met them. I would like to ask "X. Y. Z." how can a nurse properly nurse the patients in a ward, also do the other duties necessary, when she is herself ill? One can feel very ill with "only a headache" or "sore throat," &c. But "X. Y. Z.," "Scot," "Old Westminster," "H. M. G.," "Justice," and "A. L." all keep clear of the point from which the correspondence started—viz. that the twelve-hour duty at night is too much for nurses. Do they not consider it too long?

### TRAINED NURSES AND UNTRAINED WOMEN.

"A Private Nurse" writes: "St. Thomas's Nurse" recently wrote on the misuse of uniform. Will you please grant me space in your much-valued paper to give my opinion on the subject? I quite agree with a "St. Thomas's Nurse" that the uniform should be the monopoly of trained nurses, but I certainly do not think the blame lies with the medical men as a "St. Thomas's Nurse" infers. If I may say so, I think that it is putting a very low estimate on trained nurses to suggest that a doctor is only able to tell the difference between the trained and untrained by asking to see their certificates. Surely the woman who has received three or four years' training in a hospital will carry out her duties in attendance upon the sick in a different manner to the woman who has only received a few months' training, or none at all. If this is not the case why, then, do we train? Theory is absolutely necessary to make a good nurse, but if we are unable to put our theory into practice, then I say we are of little use as nurses.

"Live and Let Live" writes: Will you kindly allow a "fever nurse" space in your valuable paper to say a few words in reply to "A St. Thomas's Nurse"? While I certainly think that nursery-maids should not be allowed to wear nurses' uniforms, I do not see why fever nurses should be denied that privilege if they wish to do so, either for economy or for any other reason, or why fever nurses should be looked down upon so by their more fully trained sisters, for after all a "fever" nurse is as much a nurse and as indispensable as a St. Thomas's nurse. Neither does the "want" of general training make a woman a disgrace to her sex. It is not training or non-training that makes a woman's moral character, and is it "only" fever nurses who get up to those "pretty pranks" which "A St. Thomas's Nurse" speaks of? "A St. Thomas's Nurse" would not like all trained women to be condemned on account of the behaviour of one or two. With respect to the masseuse who gained her certificate in a fortnight, it is quite evident that some could take in as much in that time as it would take others three months to do. And surely no training school would grant a certificate to an incompetent person.

"One Who Knows" writes: With reference to a letter written a fortnight ago by "A St. Thomas's Nurse" on untrained women and fever nurses, the writer is evidently writing without having had any experience in fever nursing, or she would know that fever nurses are as essential to that branch of the profession as maternity nurses are to theirs. How many general trained nurses are there who have never had any experience in fever work, who consequently know as little about the complications of the various fevers as untrained women (as she calls fever nurses) do about surgical work. As matron of a fever hospital I have to deal with general and fever nurses, and always find a trained fever nurse vastly superior to a general nurse when on duty with acute infectious cases. There are, I have found, good and bad of each, and quite as many general trained nurses are a disgrace to womanhood as fever nurses. I think it was a very unkind letter for a nurse to write about her sisters, who run a greater risk of losing their lives than ever she did at St. Thomas's Hospital. Could she face small-pox, typhus, or plague as fever nurses do, thoroughly understanding their work, too? I am writing from experience gained as staff nurse in one of the Liverpool fever hospitals, and some of my fellow workers there are sisters now in general hospitals, and loved and respected in their work. Still, they were fever nurses once, and womanly women then as they are now. Would it not be kinder of the "St. Thomas's Nurse" to look at home, and see why these women are taking her bread. As to it being bad form to wear uniform, well, that is too ridiculous to discuss. It is noteworthy that the grumblers of the past few weeks about overwork, etc., are all general trained nurses.

### Appointments.

**[No charge is made for announcements under this head, and we are always glad to receive and publish appointments. The information, to insure accuracy, should be sent from the nurses themselves, and we cannot undertake to correct official announcements which may happen to be inaccurate. It is essential that in all cases the school of training should be given.]**

Almondsbury Memorial Hospital.—Miss Florence M. Smith has been appointed matron. She was trained at Bristol Royal Infirmary, where she has since been ward sister and temporary night sister. She holds the certificate of the Central Midwives Board.

Bradford Incorporated Nurses' Institution.—Miss Dane has been appointed matron. She was trained at the Western Infirmary, Glasgow, and has since been night superintendent at the Royal Victoria Hospital, Belfast; assistant matron at the Cork Street Hospital, Dublin; and assistant matron at Highfield Infirmary, Liverpool.

Harrison Home for Epileptics, Maghull, near Liverpool.—Miss Flora M. Gibson has been appointed assistant matron. She was trained at the Great Northern Central Hospital, London, and has since been sister at the Grimsby and District Hospital. She has also done private nursing.

Hospital for Infectious Diseases, Woodbridge, Guildford.—Miss Sarah Dutton has been appointed matron. She was trained at Manchester Fever Hospital, and has since been matron of the Infectious Hospitals at St. Albans and at Croydon.

Inverness District Asylum.—Miss B. Fraser has been appointed assistant matron. She was trained at Aberdeen Royal Infirmary and Aberdeen Royal Asylum. She has since been staff nurse at Montrose Royal Infirmary, staff nurse at Aberdeen Royal Infirmary, and charge nurse at Aberdeen Royal Asylum.

King's College Hospital, London.—Miss Mary E. Ray has been appointed sister matron. She was trained at King's College Hospital, and afterwards served as ward sister. She has since been assistant superintendent at Leeds General Infirmary, and matron of Lincoln County Hospital.

Liverpool Ladies' Charity and Lying-in Hospital.—Miss Janet E. Rogers has been appointed matron. She was trained at St. Thomas's Hospital, London, and in midwifery at the British Lying-in Hospital, London. She has since been sister at the Devon and Exeter Hospital, temporary matron at the Gordon Hospital, London, has served on the Army Nursing Service Reserve in South Africa, acting as superintendent for seventeen months, and has taken private midwifery and monthly cases, gaining experience in hospital administration at the Brompton Hospital for Consumption. She holds the certificate of the Central Midwives Board.

Macclesfield General Infirmary.—Miss L. C. Gibbon has been appointed matron. She was trained at King's College Hospital, London, and the Evelina Hospital for Children, London. She has since been sister at Salop Infirmary, Shrewsbury; night superintendent at South Devon and East Cornwall Hospital, Plymouth; and assistant matron at the Royal Infirmary, Preston.

Newington Workhouse.—Miss Ellen Partner has been appointed superintendent nurse. She has been staff nurse at Southwark Union Infirmary, and has since been charge nurse under the Metropolitan Asylums Board, and superintendent nurse at Solihull Union, Colchester Union, King's Lynn Union, and Northampton Union.

North Staffordshire Infirmary and Eye Hospital.—Miss F. M. Withers has been appointed staff nurse. She was trained at Salop Infirmary, Shrewsbury, and has since done private nursing.

Ripon Cottage Hospital.—Miss E. A. Dewhurst has been appointed staff nurse. She was trained at Scarborough Hospital and Dispensary, and has since been staff nurse at Great Yarmouth Hospital and Llanelly Hospital. She has also been Queen's nurse in Manchester and has done private nursing.

Victoria Hospital, Keighley.—Miss Emily Denton has been appointed sister of the male wards and operation theatre; Miss Amy Cracknell, district nurse; and Miss Margaret Dawbarn, staff nurse. Miss Denton was trained at the Victoria Hospital, Keighley. For some time she was engaged in private nursing in Leeds. Since then she has filled the post of sister-in-charge of the district, Keighley. She holds the certificate of the Central Midwives Board. Miss Cracknell was trained at the All Hallows Hospital, Ditchingham, and at the Scarborough Hospital and Dispensary. She has since held the post of charge nurse in the Isolation Hospital, Norwich. She has also done private nursing in Norwich and in Keighley. Miss Dawbarn was trained at the Oldham Infirmary.

### Novelties for Nurses.

(By Our Shopping Correspondent.)

### THE COVENTRY CHALLENGE CYCLES.

(Messrs. Edward O'Brien, Limited, Cycle Dealers, Coventry.)

It is quite impossible for a firm to spend large sums on advertising and then sell an equally good thing at the same low price as can be charged for an article which depends for its sale upon merit alone. This fact can be at once proved by nurses who purchase the Coventry Challenge bicycle. All the money they pay they will find has been put into the machine itself. It is the only cycle in the world which is backed with a ten years' guarantee. Edward O'Brien, Limited, of Coventry, began with guaranteeing the wear of the cycle for four years, then increased it to five, and now throughout 1906 they will give a ten-years' guarantee with every bicycle which is bought from them. This fact speaks for itself. Owing to their enormous turnover, the makers are able to offer extremely advantageous terms or easy payments to nurses. Those whose bicycles are wearing out should look into this matter for themselves. They will not be disappointed.

### RUBBER HEELS AND SHOES.

(Revolving Heel Company, Preston.)

A great boon to nurses, and indeed to all whose calling necessitates standing or walking, are the Wood-Milne rubber heels. These can be procured either stationary or revolving, and while ensuring a quiet firm tread, they can be fitted on to the smartest shoe, and render worn-down heels an impossibility. The Shoeshine, produced by the same company, in both tan and black, is a great convenience, making the cleaning of one's own shoes far removed from the disagreeable task it was in bygone days. The smallest quantity should be applied with a stiff brush, and the shoe afterwards polished with a leather or soft pad.

### "HYGICS."

(Messrs. Hunter and Co., Parliament Street, Nottingham.)

The hygienic sanitary towels known as "Hygics" are a great improvement on the style of things usually supplied in this shape. They contain no cotton-wool, waste, or filling of any description. But their chief recommendation is that they will wash or burn, so that those who prefer to use washing towels will not need to purchase any other kind when going on a sea voyage, for instance. Being made of loosely knitted cotton they are very easily ignited, and have been rendered aseptic by a special process. Messrs. Hunter have designed a special belt, known as the "comfort" belt, with which these towels can be worn. They can easily be adapted for the use of infants. The prices are 18s. per gross, sample dozen 1s. 9d. post free. Comfort belt, 6d. each; larger size, 9d. A belt will be sent free with the first order of one gross "Hygic" towels.

### FOR NURSES' UNIFORM.

Messrs. Moore, of the Belfast Linen Warehouse, Albion Street, Leeds, who are already well known as specialists in all that appertains to a nurse's uniform, have sent me specimens of a large number of their commodities. I think I am most struck by the bonnets, which are all that could be wished in fitness, appearance, and quality. The "Edna" is of a good-quality blue straw, trimmed with a gathering of dark blue velvet round the front, which gives a soft and full appearance, and surmounted by a very handsome bow of dark blue velvet. The price is only 7s. 11d. The "Coronet" is a very neat little bonnet in fine black straw. A narrow coronet-shaped band of blue velvet forms the front, between which and the crown is a tastefully arranged bow of the velvet. The "Dora" has a front of blue velvet, resembling the Sister Dora cap, and is further trimmed with a twisted band of velvet between the front and the crown of the bonnet. All the bonnets are well finished and of superior appearance. Messrs. Moore have sent two very light-weight summer cloaks in dark blue and black. One has a long detachable cape, and the other a small three-lappet cap reaching to the shoulders. The price is moderate. Then I have before me some very nice aprons, especially two made in nainsook, with bib and shoulder-straps. There are others of stronger material, and all are well fashioned. The prices range from 2s. 6d. to 3s. 6d. I would draw attention to the cuffs and collars, which are offered at very reasonable prices. The "Queen" cuff is very attractive, and of good value at 7s. 9d. per dozen. All kinds of bonnet-strings are to be had at most moderate price, also very nice washing belts and dainty Red Cross pincushions to attach to the chatelaine. Caps of all kinds and linen over-sleeves are very inexpensive and good in quality.

### SERGES FOR NURSES.

(Messrs. Egerton Burnett, Limited, Royal Serge Warehouse, Wellington, Somerset.)

A bundle of patterns from Messrs. Egerton Burnett, Royal Serge Warehouse, Wellington, Somerset, always fills one with mingled feelings of satisfaction and despair—satisfaction that the very moderate prices of the materials bring them within the scope of a scantily filled purse; despair at having to decide between the conflicting merits of such numbers of attractive materials, each of which is certainly the prettiest and most suitable till—one takes up the next one! The serges from Messrs. Egerton Burnett's warehouse are, of course, world-famous, and nurses' cloaks can be made to order in any shape required from 23s. 11d. The serges are of varying colours and degrees of thickness, and are "waterproofed." Uniform dresses can be made to order in washing materials from 15s. 11d. The patterns of these cottons are most tempting, and present great diversity of style and colour. The plain cottons are a very pleasing feature, and are always popular. Costumes and blouses are made to order at the most surprising figures. A skirt for 10s., and an entire costume for 26s. fill one with amazement. The patterns of tweeds are particularly good, and since tweeds have become so fashionable of late it is useful to know that a skirt can be had for 20s. 6d. and a costume for 52s. 6d. White serges have a charm of their own—they wash so well, look so fresh and pretty, and yet supply the warmth that in our climate is often so necessary. A costume in a very pretty fancy serge can be had for 38s. 6d., and a skirt alone for 15s. 9d. White lining for these serge dresses, if required, can be had for 9d. a yard, width 40 inches. Some of the striped serges and flannels are very neat, and a striped material always seems to last clean longer than a plain one. Skirts suggest underskirts, and there are some very pretty patterns of moirettes, very stout and strong, at 12s. 6d. Moreen underskirts can be had in all shades at 8s. 9d. Those who indulge in cycling will be glad to hear of the "Wontarewilware" suiting, which is specially adapted for hard wearing and noted for its durability. The width is 54 inches and the prices 4s. 11d. and 5s. 11d. a yard. It is when the fascinating assemblage of drills, linens, zephyrs, delaines, and silks are contemplated that despair gets the upper hand of satisfaction. It is clearly impossible to make purchases of all, much as one yearns to, and yet how to select is a matter of the utmost difficulty. There are such pretty shades of linen at only 1s. 3d. a yard, 36 inches wide. The fancy spot cambrics at 11½d. a yard would make the daintiest blouses. The striped and checked zephyrs at 7¾d. and 6d. a yard are very charming. A distinct novelty in zephyrs is a silk weft zephyr at 1s. 5d. a yard; this is made up in a variety of delicate shades, and has a very soft and silky appearance. Delaines are perhaps among the best investments for possessors of a limited wardrobe. There are some attractive sprigged patterns at 1s. 6½d. a yard, some striped floral designs at 1s. 8d., and a stylish-looking delaine, embroidered in different colours, at 2s. 3d. a yard. For summer dresses nothing could be more charming than "The Elise" at 1s. a yard, a silky fabric with a most artistic pattern. Forsaking with reluctance the bewildering variety of spring and summer materials, mention must be made of some wonderfully cheap picnic rugs at 3s. 11d. each, size 48 by 66. They will be found very useful by nurses who delight to spend their "off duty" in the garden, when such a privilege is available. "The Shrinknaught," an unshrinkable fabric for dressing-gowns or jackets, will be found to supply a felt want; it is 1s. 9d. per yard, 31 inches wide, and is made in a good variety of shades and patterns.

### A Book and its Story.

### RENOUNCEMENT.*

"The Shadow of Life" is the clever study of a man, Gavan Palairet, gifted to the point of genius. But his childhood was shadowed by too early a realisation of the irony and sadness of life through the daily spectacle of the sufferings of a loved, and beautiful mother, owing to the neglect of a dissipated father. He is sent, as a boy, from India to the care of some relations—an old soldier grandfather and two maiden aunts—living on their estate in Scotland. Here also is an orphan girl cousin, Eppie Gifford. She stands in strong contrast to the sensitive, intellectual boy who arrives one spring day at the hospitable but bleak looking house on the moors from the far-away East, with its colour and glamour. Eppie has been awaiting his arrival for some hours. "It was a very jaded traveller that she saw. . . . The General bundled out of the fly and handed rugs, dressing cases, and cages to the maid, making a passage for Gavan's descent. . . . Gavan as he ascended the steps seemed at once weary, frightened, and composed. He had a white thin face and black hair; the sort of face and hair, Eppie thought, that the wandering prince of one of her own stories, the prince who understood the rook's nest, might have had. . . . The eager welcome she had in readiness for him seemed out of place before his air of gentle self-possession, going as it did with the look of almost painful shrinking. . . . The sad boy was frozen and he chilled others." As his shyness wears off, after the first few days, he becomes more at ease with his new relatives. Eppie and he become chums and have long rambles together. "It was in the wanderings over the moors and in the birch woods and up the hill-side where Eppie took him to see her views that the bond really drew to closeness. Here nature and little Eppie seemed together to thaw him, to heal him, to make him unconsciously happy. A fugitive colour dawned in his wasted cheeks; a fragile gaiety came to his manner. . . . Gavan talked a great deal, quickly, with a sort of nervous eagerness. There grew in Eppie's mind a vast mirage-like picture of the strange land he came from; the great mountains about their high summer home; the blue shadowed verandahs; the flowers he and his mother grew in the garden; the rides at dawn; the long hot days; the gentle softly moving servants, some of whom he loved, and told her a great deal about. Then the crowds, the swarming colours of the Bazaars in the great cities. 'No, no, don't wish to go there,' he said . . . his head bent, his eyes looking before him. . . . I hate it . . . I hate the thought of anyone I care about being there. . . . It is vast and meaningless, I can't describe it to you; you expect all the time to wake up and find nothing.'"

Shortly afterwards Gavan goes to a public school and later to Oxford. At each place he acquits himself with distinction. An eccentric cousin, who had taken a fancy to him, dying just as he left Oxford, left him a small but well endowed estate. Then his mother comes to England and he is able to give her the home that he had been longing to offer her. But Gavan since his boyhood had changed materially. Morbidly sensitive to the suffering that he saw on all sides and finding no adequate solution to its mystery in the Faith which in earlier years he had held in reverence, he had drifted after an exhaustive study of Kant, Spipoza, Schopenhauer, and other unorthodox writers, into agnosticism. To a man of his temperament such a result could only wreck interior happiness. "Religion had meant too much to him for its loss to be the merely disturbing epoch of readjustment that it is in much young development. He found himself in a reeking horror of darkness, where the only lights were the dim beacons of science and the fantastic will-o'-the-wisps of æstheticism. In the midst of this chaos he saw his mother again." He had dreaded the meeting. How could he see her and hide the inner desolation? "He found her when she came, sadly changed, worn with suffering, fading visibly, and 'drooping like a fading flower.' How could he add to her sorrows by telling of his spiritual desolation? . . . He took her to the quiet old house, among its hedges, its high walled gardens and deep woods. He gave her all that it was now too late to give—beauty, ease, and love."

There is an undercurrent of bitterness, of revolt against destiny, of despair, in Gavan's mind, accentuated by the thought that having resigned the Christian's hope of immortality, he is unable to set at rest the questionings, arising unceasingly, by the mere negations which he has set up in the place of the eternal future promised by it. So, unable to soothe his mother's latter hours by the consolations of religion, her coming, which should, even in her disabled state, have been a source of thankfulness to him, is spoken of thus: "With the good fortune came the bitter irony that turned it to dust and ashes." For all his affection could not stay the hand of death; it could only help her to die in peace, solaced by hopes which he no longer shared. "She died at last and Gavan lived through all that followed in a stupor. Conscious of a dual personality within himself he leads the life of a scholarly man of the world, known and admired by all, understood by the few. At Oxford: "There was the Gavan of the river, the debate, the dinner, popular among his fellows, gentle, debonair, already the man of the world through the fineness of his perception, his instinct for the fitting, his perfection of mannerless manner that was the flower of selflessness. And there was the Gavan of the inner thought, fixed always in its knot of torturing perplexity. To the inner Gavan the Gavan of human relations was a wraithlike figure. . . . Those who knew him better found him charming and perplexing. He seemed to have no barriers, yet one could not come near him. His centre receded before pursuit—and he was much pursued. His exquisite head, the chill sweetness of his manner, the strange piercing charm of his smile drew eyes and hearts to him. Idly amused he saw himself, all inert, hoisted from step to step, saw friends swarm about him and hardly an enemy's face."

It is wished by many of his friends that he should take up politics, but he has no vocation for political life, and he goes out to India as secretary to the Viceroy—and an episode marks this period which he treats characteristically. A few years later he is on his way to England again. He goes very soon to Scotland to visit his early home and his old friends there, from whom, through the impelling force of circumstances, he has been separated from boyhood. "He had never seen Eppie again, and sixteen years had passed. It was this that Gavan was thinking as the Scotch express bore him northward on a dark October night." He hears rumours of the Eppie that had developed from the one of past years. "Poor little Eppie of childhood; she was lost for ever. But all the clearness of the night concentrated at dawn into the vivid memory of the past where they had wandered together, sharing joy and sorrow." What of joy and sorrow this meeting brings to each must be seen in the book itself. "The Shadow of Life" is not a book for all readers, but to those to whom it appeals it will, in spite of its sadness, be welcomed for its intellectuality and originality.

* "The Shadow of Life." By A. E. Sedgwick. (Constable, 6s.)

### Death in our Ranks.

The Somerset County Nursing Association has recently sustained a great loss by the death of Miss Elizabeth Tite, better known as "Nurse Lizzie." She was for many years a Queen's Nurse, and was trained in midwifery at Glasgow, in district work at Lincoln, gained surgical experience at the London Hospital, and was nurse for seven years and afterwards head nurse at the Hahnemann Home for Convalescent and Chronic Cases at Bournemouth. In 1892 she became district nurse for the villages of Spaxton, Over Stoney, and two other villages in Somersetshire, where, except for occasional absences for illness, the result of tubercular mischief, she worked till her death. She was beloved by all amongst whom she ministered, and her great wish that her work should end only with her life was granted her, for her last illness was very brief. The Vicar of Over Stoney, in preaching an "In Memoriam" sermon the Sunday after her death, at the age of forty-eight, said that "She left a blank which he felt could never really be filled again."

### Where to Go.

Easter Excursions on the Great Central Railway from Marylebone Station to all the principal towns and holiday resorts in the Midlands, North of England, North-East and North-West coast watering-places, Scotland, and Ireland, Wednesday, April 11, and Thursday, April 12. An A B C programme, showing all particulars at a glance, can be obtained free at Marylebone Station, or at any of the company's suburban stations.

### TRAVEL NOTES AND QUERIES.

By our Travel Correspondent.

Accommodation in Malvern (May).—Mrs. Hathaway, Aldwyn Tower. Mrs. S. E. Matthews, Malvern House Private Hotel. Mrs. Baker, Scarborough Boarding Establishment. Miss Trent, Wellington Hotel. The cheapest of these is Mrs. Baker's house, which offers accommodation from 31s. 6d. Malvern is an expensive place. If you wish for something cheaper go for a night to one of the above addresses and look about for rooms which abound.

Rules in Regard to Correspondence for this Section.—All questioners must use a pseudonym for publication, but the communication must also bear the writer's own name and address as well, which will be regarded as confidential. All such communications to be addressed "Travel Correspondent, 28 Southampton Street, Strand." No charge will be made for inserting and answering questions in the inquiry column, and all will be answered in rotation as space permits. If an answer by letter is required, a stamped and addressed envelope must be enclosed, together with 2s. 6d., which fee will be devoted to the objects of "The Hospital" Convalescent Fund. Ten days must be allowed before an answer can be published.

### USEFUL PAMPHLETS FOR NURSES.

District nurses in particular, and all those whose work leads them amongst girls and young women, will probably be glad to know of four little books written by Mrs. Hill, Christ Church Vicarage, Pendlebury, Manchester, from whom they may be obtained direct. They are called "Homely Talks with my Girl Friends." The first is "A King's Daughter" (1d.), the second "A King's Daughter Engaged" (1d.), the third "A King's Daughter Preparing for Marriage" (2d.), and the fourth "A King's Daughter Married" (2d.). The last is for married women only. In each booklet matters of which girls and women should not be ignorant, but which from feelings of delicacy are often never talked about in the right way, are treated in a simple, straightforward manner, which should go far—if the little volumes are placed in the right hands—to increase the health of the girls of our land both physically and morally, and make them better women and better wives.

### Notes and Queries.

**REGULATIONS.**

**The Editor is always willing to answer in this column, without any fee, all reasonable questions, as soon as possible.**

**But the following rules must be carefully observed.**

**1. Every communication must be accompanied by the name and address of the writer.**

**2. The question must always bear upon nursing, directly or indirectly.**

**If an answer is required by letter a fee of half-a-crown must be enclosed with the note containing the inquiry.**

*Roman Catholic Guild.*

Several correspondents replying to the inquiry of "E. H.," send us the address of the Catholic Nurses' Guild. Communications should be addressed to the Sister Superior, Convent of the Visitation, Harrow-on-the-Hill.

*Paint for Hospital Walls.*

(1) I am anxious to find out what is the paint generally used for hospital walls which are glazed, when they can be washed down without spoiling the polish or walls, and always look clean.—*Matron.*

Best ivory-white enamel.

*Boston and New York.*

(2) Are there fever hospitals in America, say New York or Boston, where they take assistant nurses as they do here, not having had experience in a general hospital? I have had two years' fever training, and four years as an assistant nurse. Would I be likely to do as well there as here? Also, what American paper, with advertisements, is similar to The Hospital, and where could I get it?—*A. C.*

You would find it very difficult, if not impossible, to get the position you wish in America. But you might advertise in the *American Journal of Nursing* or *The Trained Nurse*, published monthly. Write for particulars to the manager of the Scientific Press, 28 Southampton Street, Strand, W.C.

*Cairo and the South of France.*

(3) Will you give me the address of a good nursing institution at Cairo, also one or two in the South of France—Cannes preferred?—*Cavendish.*

The Kasr-el-Ainy Hospital, Cairo; The British Hospital, Sunny Bank, Petit Juas, Cannes; The Nursing Institute, Villa Pillatte, Avenue Désambrois, Nice.

*Nursing in Madrid.*

(4) Is there any prospect of getting employment as a nurse here in Madrid? I have been nursing for the past six years in Ireland, and hold my certificate from St. Vincent's Hospital—two years in the wards, medical, surgical, and fever.—*A. M. O'T. de L.*

As you are on the spot, you had better make inquiries at the British Consulate.

*Massage.*

(5) Can you give me any information regarding the fees that are charged for instruction in massage and medical gymnastics?—*M. S. M.*

Write for particulars to the Matron, School for Massage, National Hospital for the Paralysed and Epileptic, Queen Square, Bloomsbury, W.C., or advertise or answer advertisements.

*Homes and Dispensing.*

(6) I should be glad if you can tell me the names of Homes for the Dying in London and Manchester, and of a college in London suitable for the study of dispensing; also the best book for the first course of study of the same.—*A. P.*

Free Home for the Dying, 29 North Side, Clapham Common, S.W.; Friedenheim Hospital, Upper Avenue Road, Swiss Cottage, N.W. Mr. James Ferguson, Secretary to the Northern Counties Hospital for Incurables, Manchester, may be able to supply you with the information you require about Manchester. Write to the Pharmaceutical Society, 17 Bloomsbury Square, W.C., for advice as to dispensing.

*Register of Cases.*

(7) Will you kindly advise me? In accordance with the rules of the Central Midwives Board, I keep a register of cases, which is duly inspected by the authorised inspectors of midwives. The Superintendent of an institution for nurses to which the Association employing me here is in some way affiliated now demands for her inspection my own register of cases as well. What is the correct thing to do?—*District Midwife.*

It depends on the terms of your agreement and the custom of the Association employing you. But from your letter we should gather that probably the Superintendent is within her rights in demanding to look at your book.

*Training.*

(8) Is a certificate received in Brompton Hospital, London, as good as one received in a general hospital?—*M. S. T. R.*

However good the training may be in a special hospital to obtain a post as Sister in our large hospitals, or in the Army Nursing Service, it is necessary to have three years' consecutive training in a large general hospital.

(9) I am 22 years of age, and should like very much to learn to be a nurse, but I have not any money by me to pay for learning. I would willingly give my time for learning.—*Miss T.*

Most of the hospitals will receive you, if suitable, without any premium. But you cannot enter before 23 years of age, though it is not too early to send in your application. Consult "How to Become a Nurse," published by the Scientific Press, Limited, Southampton Street, Strand.

(10) Can you tell me if a nurse who has had three years' general training at the B —— Infirmary can be considered a thoroughly qualified nurse? Is the training there as good as that obtained in a larger place?—*Gee-Gee.*

Probably the training is quite as good, but as the Infirmary you mention contains less than a hundred beds, you would not be qualified for the higher posts in the nursing world.

*Army Nursing Service Reserve.*

(11) Can you tell me what advantage a nurse has if she joins the Army Nursing Service Reserve? Would it make any difference as to getting into an infirmary or hospital? Also, in the event of being on service abroad, would a nurse have any difficulty in getting into a civil general hospital?—*An Inquirer.*

One of the advantages of belonging to the Army Nursing Service Reserve is that you would be amongst the first to go out to nurse the soldiers in time of war. The fact of your being a member would not probably affect your chances of obtaining a post in an infirmary or hospital either before or after.

*Dispensing.*

(12) I hold a certificate for dispensing gained at the examination in connection with the Apothecaries' Hall, and am now desirous of getting a post as assistant-dispenser in a hospital. I should be willing to give any spare time to work as a probationer. Could you advise me?—*B. O.*

Watch the advertisement sheets of the medical papers, and advertise yourself.

*Compensation for Maternity Nurses.*

(13) A lady engaged me last month to nurse her during her confinement in the summer. She said in her letters that she should require me at least six weeks, and agreed to pay me all the fees I asked, which were rather high ones. On account of this I have refused other offers of work for that time. Last Friday I received a card to say that the lady had had a miscarriage, and will not want me at all. Can I claim any compensation? There was no proviso made for this occurrence. —*L. O. S.*

You should have made arrangements for contingencies when you settled the terms with the lady. All maternity nurses encounter these difficulties now and again. If you write to the lady she may be willing to give you half fees, a course which is generally followed in such cases.

(14) My wife engaged a maternity nurse to come to her about the end of January. As a matter of fact the baby did not arrive until February 12, so the nurse's services were not required, and she did not arrive at the house until that date. The nurse has now sent me in a bill for the two weeks between January 29 and February 12 at £2 2s. per week for the month —I was to pay £8 8s. I may say that the nurse left after she had been with us a fortnight because she quarrelled with an old servant of ours. She is nursing on her own account. Is there a custom to pay if the baby does not arrive when it is expected? My doctor says not, and that the nurse takes the risk.—*Inquirer.*

The compensation due to a maternity nurse who may not be required at the time stated should be settled always at the time of engagement. It is customary for the nurse to charge from the time she is engaged. She could not, of course, engage herself for that time to anyone else.

**Handbooks for Nurses.**

| | Post Free. |
|---|---|
| "How to Become a Nurse: How and Where to Train." | 2s. 4d. |
| "Nursing: its Theory and Practice." (Lewis.) ... | 3s. 6d. |
| "Nurses' Pronouncing Dictionary of Medical Terms." | 2s. 6d. |
| "Complete Handbook of Midwifery." (Watson.) ... | 6s. 4d. |
| "Preparation for Operation in Private Houses." ... | 0s. 6d. |

Of all booksellers or of The Scientific Press, Limited, 28 & 29 Southampton Street, Strand, London, W.C.

### For Reading to the Sick.

### AFTERWARDS.

O heart that, sad and weary,
 Dost count thy load too great,
Thy night too dark and dreary,
 Thy way too desolate;
Take comfort in Thy sorrow,
 God sets an end to woe;
There comes a happy morrow,
 A day thy Lord doth know.

Not clear nor dark that morning,
 That time not day nor night;
Peace broods upon its dawning,
 Secure and infinite.
It sees no cloud o'ercasting
 Its sunshine evermore;
No tears, no pain, no fasting,
 The vigil eve is o'er. *Anon.*

Even in this world, among all the perishing things of earth, a life which struggles through difficulties and nobly does its duty when, as in Job's case, the hand of the Lord is upon it, and when friends fail, such a life is in a sense even here below imperishable. As no fashion can alter its standing-point, so can nothing affect its worth; its memorial shall not perish with it, but it shall bear for ever the reflection of that true Light which lighteth every man that cometh into the world.

All thoughtful persons would admit readily that God's gifts are manifold. Life and breath and all things are from Him, given to us by His loving kindness and intended for our highest good. But in the spiritual life we require to look much beneath the surface to arrive at anything like the true value and meaning of His gifts.

God alone can fit the comfort to the need; His loving kindness is far tenderer than man's. David knew this when he said, "In the time of trouble He shall hide me in His pavilion; in the secret of His tabernacle shall He hide me. He shall set me up upon a rock." Here we are shown two opposite ways of God's dealings towards His troubled servants—"Hiding" and "setting up." How welcome is the first, and how glorious will be the full realisation of the second promise!—*Mrs. Campbell.*

"He that overcometh will I make a pillar in the temple of my God." As we read these words, what an image of stability and of rest rises before us; a pillar, firm and immovable, in the everlasting Temple. Oh! what a contrast to the earthly state of these trembling children of God! Here the tossings to and fro, the yearnings and unrest, the shadows in the sunshine, and the bitterness of joy; there the rest and the security; the unutterable peace of God; the heavenly seal which marks them for his own; the name of the Blessed written upon them: a new name, the beginning and earnest of a new life. "And they shall go no more out," they shall dwell for ever in His Temple, in the Home of His Love; and their joy shall be full, for they shall be with Him.—*M. E. Townsend.*

"I know not the way I am going,
 But well do I know my Guide;
With a childlike trust I give my hand
 To the Mighty Friend at my side.
The only thing that I say to Him,
 As He takes it, is, 'Hold it fast!
Suffer me not to lose my way,
 But bring me home at last.'" *Anon.*

## Figures and Tables

**Figure f1:**
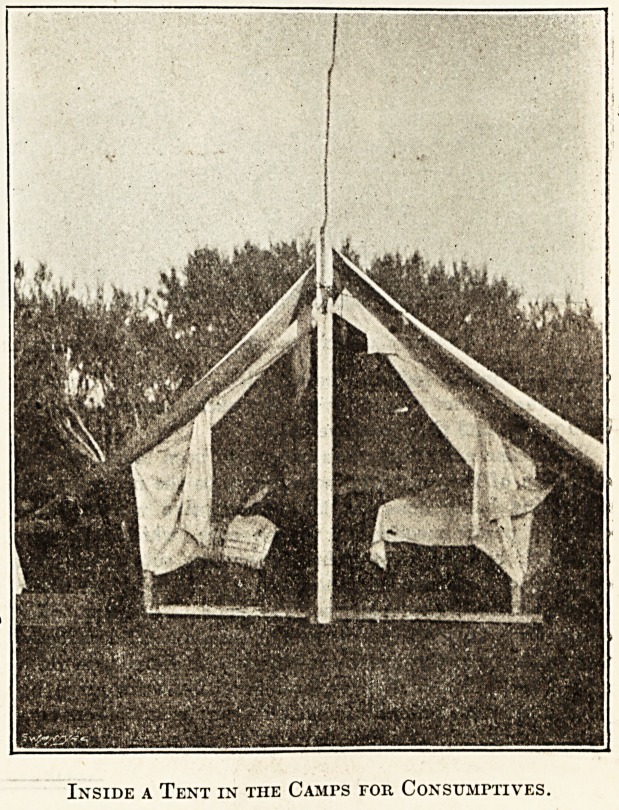


**Figure f2:**